# Robust synchronization of the cell cycle and the circadian clock through bidirectional coupling

**DOI:** 10.1098/rsif.2019.0376

**Published:** 2019-09-11

**Authors:** Jie Yan, Albert Goldbeter

**Affiliations:** 1 Center for Systems Biology, School of Mathematical Sciences, Soochow University, Suzhou, People's Republic of China; 2 Unité de Chronobiologie Théorique, Faculté des Sciences, Université Libre de Bruxelles (ULB), 1050 Brussels, Belgium

**Keywords:** cell cycle, circadian clock, coupled oscillators, synchronization

## Abstract

The cell cycle and the circadian clock represent major cellular rhythms, which appear to be coupled. Thus the circadian factor BMAL1 controls the level of cell cycle proteins such as Cyclin E and WEE1, the latter of which inhibits the kinase CDK1 that governs the G2/M transition. In reverse the cell cycle impinges on the circadian clock through direct control by CDK1 of REV-ERBα, which negatively regulates BMAL1. These observations provide evidence for bidirectional coupling of the cell cycle and the circadian clock. By merging detailed models for the two networks in mammalian cells, we previously showed that unidirectional coupling to the circadian clock can entrain the cell cycle to 24 or 48 h, depending on the cell cycle autonomous period, while complex oscillations occur when entrainment fails. Here we show that the reverse unidirectional coupling via phosphorylation of REV-ERBα or via mitotic inhibition of transcription, both controlled by CDK1, can elicit entrainment of the circadian clock by the cell cycle. We then determine the effect of bidirectional coupling of the cell cycle and circadian clock as a function of their relative coupling strengths. In contrast to unidirectional coupling, bidirectional coupling markedly reduces the likelihood of complex oscillations. While the two rhythms oscillate independently as long as both couplings are weak, one rhythm entrains the other if one of the couplings dominates. If the couplings in both directions become stronger and of comparable magnitude, the two rhythms synchronize, generally at an intermediate period within the range defined by the two autonomous periods prior to coupling. More surprisingly, synchronization may also occur at a period slightly below or above this range, while in some conditions the synchronization period can even be much longer. Two or even three modes of synchronization may sometimes coexist, yielding examples of birhythmicity or trirhythmicity. Because synchronization readily occurs in the form of simple periodic oscillations over a wide range of coupling strengths and in the presence of multiple connections between the two oscillatory networks, the results indicate that bidirectional coupling favours the robust synchronization of the cell cycle and the circadian clock.

## 1. Introduction

Evidence connecting the cell cycle and the circadian clock was obtained in unicellular organisms such as *Euglena* [1], cyanobacteria [2–4] and *Neurospora* [5], and in plants [6], zebrafish [7] and mammals [8–13]. Transcriptome studies indicate that a large proportion of genes in mammals are controlled by the circadian clock [14,15]. Among these, a number of cell cycle genes were shown to be expressed in a circadian manner. The connection of the mammalian cell cycle with the circadian clock was first evidenced by the circadian variation in the expression of various cell cycle genes in human tissues such as oral mucosa and skin [16–18]. However, the coupling between the cell cycle and the circadian clock is not always present [19], and its strength may vary in different conditions or cell types [8].

**Figure 1.**
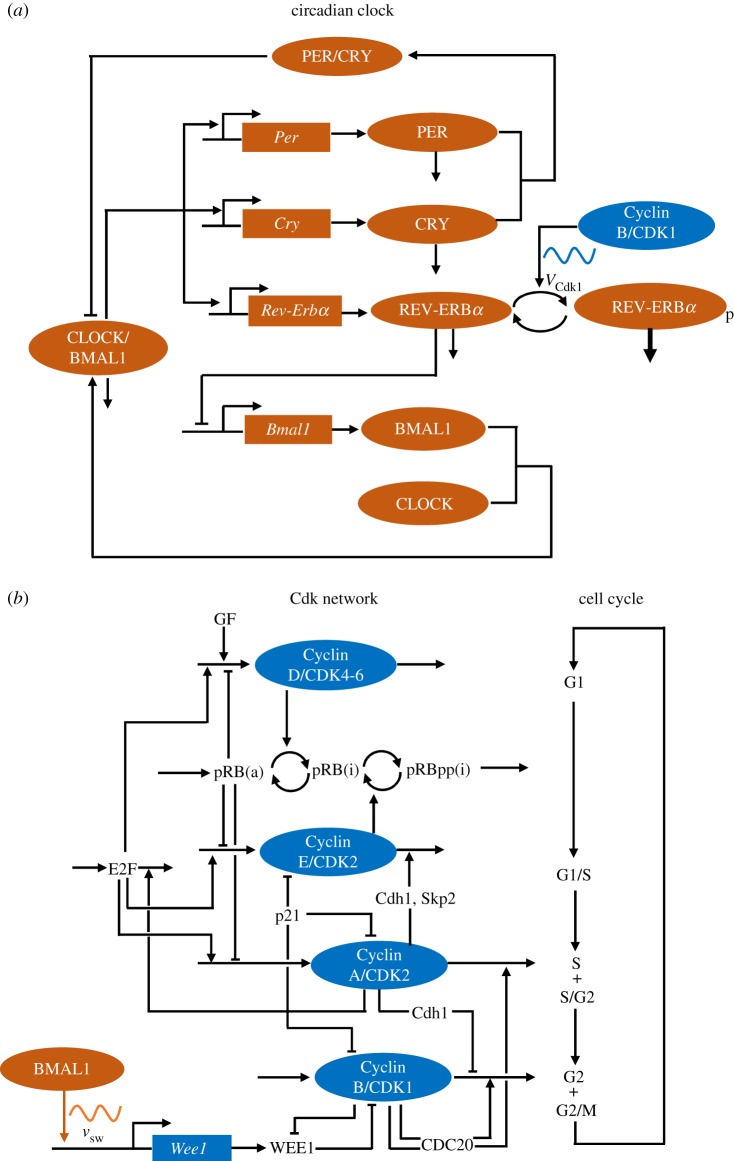
Schematic of the models for (*a*) the circadian clock and (*b*) the cell cycle in mammalian cells. In (*a*), the circadian clock network (in orange) involves the negative autoregulation of the *Per* and *Cry* genes, via the inhibition of the activators BMAL1 and CLOCK by the PER and CRY proteins. An additional negative feedback on *Bmal1* expression is mediated by the REV-ERBα protein, which is itself induced by CLOCK/BMAL1. These feedback regulations are responsible for the onset of circadian oscillations in the network (see [20–22] for reviews, and [24] for further details on the model). The cell cycle controls the circadian clock through several interactions, only one of which is shown: the phosphorylation by CDK1 (of maximum rate *V*_Cdk1_) of REV-ERBα, which enhances the degradation of this protein. In (*b*), the CDK network (in blue) that governs the dynamics of the mammalian cell cycle consists of four CDK modules, centred on the complexes Cyclin D/CDK4–6, Cyclin E/CDK2, Cyclin A/CDK2 and Cyclin B/CDK1, which control, respectively, the progression along the G1, S and G2 phases and the G2/M transition [23], as shown on the right part of (*b*). Also shown in this scheme are some of the important protein factors involved in regulation of the CDK network: growth factors (GF), the retinoblastoma protein, non-phosphorylated (pRB) or inactivated through one (pRBp) or multiple phosphorylations (pRBpp) by CDK1 and CDK2 (a, active; i, inactive); the transcription factor E2F; the CDK inhibitor p21; the proteins Cdh1, Skp2 and CDC20 involved in cyclin degradation; and the kinase WEE1, which inhibits CDK1. Only some of the main regulatory interactions are shown in this simplified scheme. The regulatory design of the CDK network is such that each CDK module activates the next module and inhibits the previous one. Such a regulation results in the repetitive, ordered, transient activation of the four CDK modules that control the successive phases of the cell cycle. The circadian clock controls the cell cycle through several interactions, only one of which is shown: the induction by BMAL1 of the expression (at a rate *v*_sw_) of the gene *Wee1*, which codes for the inhibitory kinase WEE1. The models for the cell cycle and circadian clock contain 41 and 19 variables, respectively. The models for the circadian clock and the cell cycle are described in more detail in [24] and [25], respectively. (Online version in colour.)

Experimental studies performed over recent decades have clarified the regulatory structure of the circadian clock network [20–22] and of the network of cyclin-dependent kinases (CDKs) driving the cell cycle [23] in mammalian cells. The circadian clock network involves the negative autoregulation of the *Per* and *Cry* genes via the inhibition of the activators BMAL1 and CLOCK by the PER and CRY proteins; an additional negative feedback on *Bmal1* expression is mediated by the REV-ERBα protein, which is itself induced by BMAL1 (see the scheme in figure 1*a* and [20–22] for reviews). On the other hand, the cell cycle network involves the formation of complexes between various cyclins and the cyclin-dependent kinases CDK1 and CDK2; these complexes form in turn to elicit the transitions between the successive phases of the cell cycle. The CDK network, schematized in figure 1*b*, consists of four CDK modules centred on the complexes Cyclin D/CDK4–6, Cyclin E/CDK2, Cyclin A/CDK2 and Cyclin B/CDK1, which control, respectively, the progression along the G1, S and G2 phases and the G2/M transition [23]. The CDK network is organized in such a way that each CDK module activates the next module and inhibits the previous one. Such a regulation results in the ordered, transient activation of the four CDK modules that control the successive phases of the cell cycle. Detailed computational models based on these experimental findings have been developed for the mammalian circadian clock [24,26–29] and for the mammalian cell cycle [25,30,31]. These models support the view that both regulatory networks behave as self-sustained oscillators of the limit cycle type [24–26,30,31]. The goal of this paper is to explore by means of these computational models the dynamical consequences of the coupling between the circadian clock and the cell cycle in mammalian cells.

The regulatory links between the cell cycle and the circadian clock remain to be fully characterized at the molecular level, but some information has already been gathered. Most observations so far pertain to the control exerted on the cell cycle by the circadian clock. Thus, in regenerating liver, the transcription factor BMAL1, which plays a central role in the circadian clock mechanism, induces the expression of WEE1 [32]; this kinase inhibits the cyclin-dependent kinase CDK1, which, upon binding to Cyclin B, triggers the G2/M transition. Other cell cycle genes are induced directly or indirectly by BMAL1, such as those coding for Cyclin E (a partner of CDK2) [33,34], Cyclin B [35] and p21 (a CDK inhibitor) [36]. Evidence for the reverse coupling of the circadian clock to the cell cycle comes from the synchronization of the two cellular rhythms in NIH3T3 fibroblasts [37,38]. By means of perturbations of the circadian and cell cycle periods, and by using a mathematical model of two coupled phase oscillators, Naef and co-workers [38] showed that the influence of the cell cycle on the circadian clock in these cells plays a more significant role than the reverse mode of control. All these observations indicate that, when it occurs, the coupling between the cell cycle and the circadian clock can be of a bidirectional nature.

The molecular basis for the control of the circadian clock by the cell cycle has not yet been characterized in as much detail as the reverse coupling. Such a link was recently brought to light in a study [39] showing that CDK1 phosphorylates the protein REV-ERBα, which mediates negative autoregulation of *Bmal1* gene expression in the circadian clock; phosphorylated REV-ERBα is recognized by protein FBXW7, which targets it to the proteasome. The work of Zhao *et al*. [39] thus indicates that REV-ERBα stability is modulated by CDK1-mediated phosphorylation, a view included in a recent review [10]. The question arises as to what are the dynamical consequences of such bidirectional coupling of the two cellular rhythms? Here we explore the effect of mutual coupling of the circadian and cell cycle clocks in mammalian cells by using detailed molecular models for the two regulatory networks. We determine the patterns of synchronization of the two oscillators as a function of the relative strengths of coupling in both directions.

While the circadian clock is generally considered to be a self-sustained oscillator of the limit cycle type [24,40,41], the question arises as to whether the cell cycle may also be viewed as a cellular rhythm. This view plays a central role in the present study and therefore warrants some explanation. It is based on our previous investigations of the dynamics of a detailed computational model for the CDK network driving the mammalian cell cycle [25,30,42]. As schematized in figure 1*b*, the model consists of four CDK modules centred on the complexes Cyclin D/CDK4–6, Cyclin E/CDK2, Cyclin A/CDK2 and Cyclin B/CDK1, which are tightly coupled through multiple modes of regulation involving Cyclin synthesis and degradation, CDK control by protein inhibitors and reversible phosphorylation [23]. The analysis of this model showed that, above a critical level of growth factor, the regulatory wiring of the CDK network ensures its temporal self-organization in the form of sustained oscillations of the limit cycle type, which correspond to the transient, periodic and sequential activation of the different Cyclin/CDK complexes that control the transitions between the successive phases of the cell cycle [25,42]. Similar conclusions were obtained in the presence of checkpoints [25,42]. Based on these results, the transition from cell cycle arrest to cell proliferation can be viewed as a switch, beyond a bifurcation point, from a stable steady state of the CDK network to sustained CDK oscillations, once the balance between factors that promote or impede progression in the cell cycle is tilted, beyond a threshold, towards cell proliferation [31]. Factors that contribute to passing this threshold include: high levels of growth factors, high levels of oncogenes, low levels of tumour suppressors, low levels of CDK inhibitors, high stiffness of the extracellular matrix and low cell density [25,31,42].

Positive feedback loops, associated with bistability, abound in the regulation of the cell cycle, and the role of bistability in rendering the transitions between successive cell cycle phases irreversible has been repeatedly emphasized [43–48]. The occurrence of bistable transitions has led to the view of the cell cycle as a sequential machine rather than as an autonomous clock [43,46]. The two views of the cell cycle, referred to by Murray and Kirschner [49] as *dominoes* versus *clock*, can be unified, particularly when the oscillations are associated with all-or-none transitions in CDK activity that readily arise from bistability. In our view, the observation of bistability in isolated CDK modules of the cell cycle fits with the occurrence of CDK oscillations in the whole CDK network. The link between bistability and oscillations can be illustrated by the early cell cycles in amphibian embryos. *In vitro* experiments in which the level of Cyclin B is controlled in *Xenopus* egg extracts led to the observation of a hysteresis loop, associated with the coexistence of two stable steady states, when measuring the activity of CDK1 upon increasing and then decreasing Cyclin B concentration [50,51]. This bistability results from dual positive feedback due to activation by CDK1 of the phosphatase CDC25, which activates CDK1, and inhibition by CDK1 of the kinase WEE1, which inhibits CDK1 [50,51]. Positive feedback is also linked to negative feedback, because the activation of CDK1 leads to Cyclin B degradation [52]. When allowing the variations in Cyclin B levels due to this negative feedback and to Cyclin synthesis [53], the CDK1 module becomes able to oscillate autonomously, as it repetitively moves along the underlying hysteresis loop. While bistability is observed when the level of Cyclin B is held constant and treated as a control parameter [50,51], oscillations develop when the Cyclin B level is allowed to vary.

In the mammalian cell cycle, oscillations arise when linking explicitly one bistable transition to the next in the successive CDK modules that constitute the CDK network as a whole (see fig. 10 in [42]). When the CDK modules are isolated from the network, as in the experiments on the CDK1 module in *Xenopus* egg extracts [50,51], they display bistability [43,46]. However, when coupling the different modules tightly through multiple regulations, as occurs *in vivo*, the full CDK network either evolves to a stable steady state (corresponding to cell cycle arrest) or displays self-sustained oscillations. The bistability that underlies each of the CDK modules confers to the CDK oscillations their relaxation nature once the different modules are embedded and coupled within the full CDK network [25,42]. The robustness of CDK oscillations appears to be enhanced by increasing the number of positive feedback loops in the network, due to the enlargement of the ranges in which bistability occurs in the CDK modules [54,55]. The roles of bistability and oscillations, far from being mutually exclusive, may thus be reconciled in discussing the dynamics of the mammalian cell cycle. This justifies our use of a computational model for the coupling of the cell cycle and the circadian clock in which each of the two networks evolves towards a steady state or behaves as a self-sustained oscillator.

In a previous study [56] we investigated the effect of unidirectional coupling of the cell cycle to the circadian clock. By incorporating into the model for the mammalian cell cycle its coupling to the circadian clock via WEE1, Cyclin E and/or p21, we showed that the cell cycle can be entrained to a period of 24 or 48 h, depending on its autonomous period prior to coupling. Outside these domains, entrainment fails and complex oscillations occur. Here we first show that the reverse unidirectional coupling can lead to entrainment of the circadian clock by the cell cycle. We then show how bidirectional coupling readily results in the robust synchronization of the two cellular rhythms. The synchronization period depends on the relative values of the two coupling strengths, and is generally within the range defined by the autonomous periods of the two oscillators before coupling. Numerical simulations nevertheless reveal that the cell cycle and the circadian clock may sometimes synchronize at a period below or above this range. Although the incorporation of the coupling of the circadian clock to the cell cycle may seem to be only a minor change compared with the case of unidirectional coupling of the cell cycle to the circadian clock [56], we show that it has profound effects on the patterns of synchronization of the two cellular networks.

## 2. Results

### 2.1. Unidirectional coupling of the cell cycle and the circadian clock

To determine the effects of unidirectional and bidirectional coupling we follow the approach used by Gérard & Goldbeter [56] in coupling the 39-variable model for the mammalian cell cycle [25] to the 19-variable model for the mammalian circadian clock [24,26]. In this section we consider in turn the case where the cell cycle is unidirectionally coupled to the circadian clock—the only situation of unidirectional coupling that was addressed in our previous publication—and the reverse case where the circadian clock is unidirectionally coupled to the cell cycle. Bidirectional coupling will be addressed in §2.2; it will also be discussed in §2.3, where the effect of multiple modes of coupling will be considered.

#### 2.1.1. Unidirectional coupling leads to entrainment of the cell cycle by the circadian clock

In [56] we considered the situation where the cell cycle is coupled to the circadian clock through the control exerted by the circadian clock protein BMAL1 on the expression of several genes coding for proteins of the cell cycle machinery (see the scheme in electronic supplementary material, figure S1a). We showed that induction of genes coding for the inhibitory kinase WEE1, the CDK inhibitor p21 or Cyclin E can each on its own, or in combination, elicit entrainment of the cell cycle by the circadian clock. Thus, depending on the strength of coupling and on the autonomous period of the cell cycle (*T*_CC_), once it becomes coupled to the circadian clock the cell cycle period goes from *T*_CC_ to 24 h or to a multiple of 48 h. The domains of entrainment of the cell cycle to 24 or 48 h take the form of Arnold tongues: the range of *T*_CC_ values allowing for entrainment to the circadian clock period (*T*_CR_ = 24 h) starts around *T*_CC_ = 24 h and increases with the coupling strength, until no entrainment occurs when the coupling becomes too strong, because CDK1 is permanently inhibited by WEE1.

To describe the coupling of the cell cycle to the circadian clock we will primarily assume here that it only takes the form of the induction of *Wee1* by BMAL1. In §2.3 we will consider the case where Cyclin E is regulated by BMAL1, alone or in conjunction with WEE1. Thus we supplement the equations describing the cell cycle network by an equation for the time evolution of *Wee1* mRNA, in which the synthesis of *Wee1* mRNA is described by a constant term, related to basal mRNA synthesis, and by a term for *Wee1* mRNA expression dependent on BMAL1 (see Section 2 in the electronic supplementary material). This is a slightly modified form of the coupling considered in our previous publication [56], where the constant, basal rate of *Wee1* expression was incorporated only in the kinetic equation for the proteins while the induction by BMAL1 of *Wee1* mRNA synthesis was included in the kinetic equation for mRNA.

**Figure 2.**
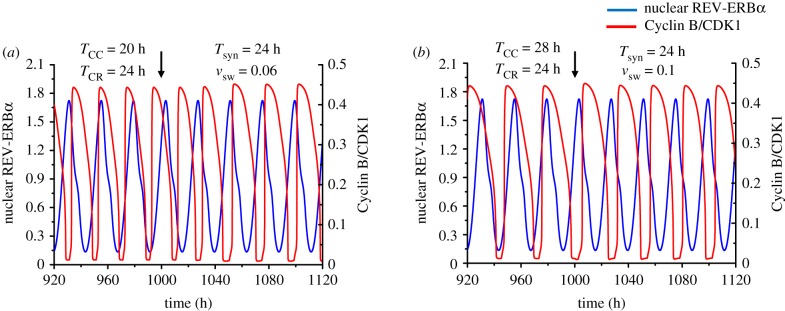
Unidirectional coupling of the cell cycle to the circadian clock. The time series show the entrainment of the cell cycle by the circadian clock when the autonomous period of the cell cycle (*T*_CC_) is 20 h (*a*) or 28 h (*b*), while the autonomous period of the circadian rhythm (*T*_CR_) is 24 h. In both cases the cell cycle can be entrained to oscillate with a period of 24 h when it is unidirectionally coupled to the circadian clock via induction of *Wee1* by BMAL1, starting at the time marked by the vertical arrow. Before coupling begins, the time series show the cell cycle and the circadian clock oscillating independently at their autonomous period. The strength of coupling of the cell cycle to the circadian clock, *v*_sw_, is equal to 0.06 µMh^−1^ in (*a*) and 0.1 µMh^−1^ in (*b*). The scaling parameter *eps* used to fix the cell cycle period is equal to 21.58 in (*a*) and 15.35 in (*b*). The blue curve represents the time evolution of nuclear REV-ERBα (a circadian variable) while the red curve represents the time evolution of Cyclin B/CDK1 (a cell cycle variable). For this and subsequent figures, parameter values are given in the electronic supplementary material. (Online version in colour.)

Although we previously examined in detail the entrainment of the cell cycle in the case of its unidirectional coupling to the circadian clock, for the sake of completeness we present in figure 2 the results of additional simulations in the case where the autonomous period of the cell cycle is 20 h (figure 2*a*) or 28 h (figure 2*b*), i.e. shorter or longer than the period of the circadian clock, *T*_CR_, which will remain fixed at 24 h throughout this paper. The results indicate that in both cases the cell cycle period shifts to 24 h: thus, the cell cycle is entrained by the circadian clock. As previously stressed [56], the result in figure 2*b* is counterintuitive. Indeed, while it is easy to understand how a cell cycle of 20 h can be slowed down to 24 h upon using WEE1, which inhibits progress in the cell cycle, it is less clear how the cell cycle can accelerate and decrease its period from 28 h to 24 h when coupled to the circadian clock via WEE1. The simulations in figure 2*b* show that this occurs through narrowing the peaks in CDK1 and CDK2 activity.

#### 2.1.2. Unidirectional coupling leads to entrainment of the circadian clock by the cell cycle

Although there is ample evidence that the cell cycle is coupled to the circadian clock, in more than one way, we may wonder whether, in the absence of such coupling, the sole coupling of the circadian clock to the cell cycle might also lead to entrainment. In such a case the circadian clock should shift to the cell cycle period. To introduce such reverse unidirectional coupling we must incorporate into the model terms describing the link of the circadian clock to the cell cycle. Recent experiments [39] indicate that CDK1 phosphorylates REV-ERBα, and thereby marks this protein for degradation via its binding to protein FBXW7. Through such a mechanism the cell cycle protein CDK1 controls the amplitude of the circadian clock [39]. Here, we take into account this observation by considering that the circadian clock is coupled to the cell cycle through phosphorylation of REV-ERBα by CDK1 (see the simplified scheme in electronic supplementary material, figure S1b). For simplicity we do not consider explicitly the binding of phosphorylated REV-ERBα to FBXW7 and assume that phosphorylation by CDK1 directly marks REV-ERBα for degradation by the proteasome (see Section 3 in the electronic supplementary material).

**Figure 3.**
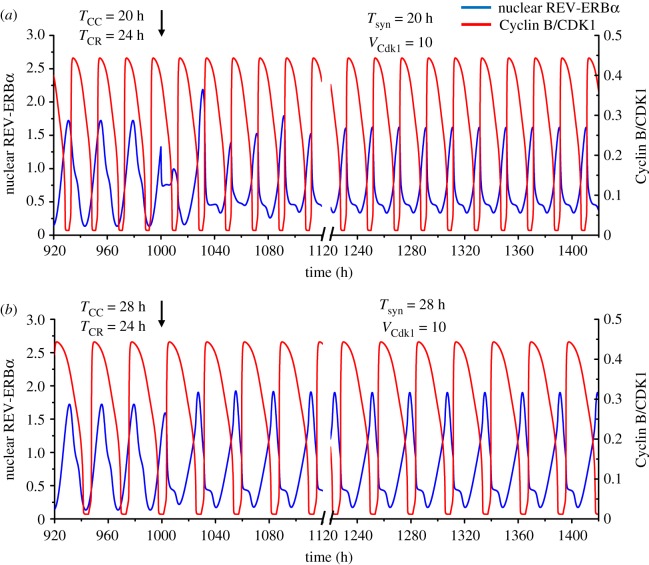
Unidirectional coupling of the circadian clock to the cell cycle. The time series show the entrainment of the circadian clock by the cell cycle when the autonomous period of the latter (*T*_CC_) is 20 h (*a*) or 28 h (*b*), while the autonomous period of the circadian rhythm (*T*_CR_) is 24 h. In both cases the circadian clock is entrained to oscillate at the cell cycle period upon unidirectional coupling via phosphorylation of REV-ERBα by Cyclin B/CDK1, starting at the time marked by the vertical arrow (*t* = 1000 h). Before coupling begins, the time series show the cell cycle and the circadian clock oscillating independently at their autonomous period. The strength of coupling of the circadian clock to the cell cycle, *V*_Cdk1_, is equal to 10 nMh^−1^ in (*a*) and (*b*), while the scaling parameter *eps* is 21.58 in (*a*) and 15.35 in (*b*). The blue curve represents the time evolution of nuclear REV-ERBα while the red curve represents the time evolution of Cyclin B/CDK1. In both panels time is interrupted between 1120 h and 1220 h to reduce the number of transients shown after the onset of coupling. (Online version in colour.)

Numerical simulations show that such unidirectional coupling can readily lead to entrainment of the circadian clock by the cell cycle when the latter has an autonomous period shorter or longer than 24 h. Thus, upon coupling of the circadian clock to the cell cycle, the period of the circadian clock shifts to that of the cell cycle when the *T*_CC_ is equal to 20 h (figure 3*a*) or 28 h (figure 3*b*). In the curves in figure 3, a 100 h interval was removed after *t* = 1120 h to reduce the number of transients shown before the coupled system displays simple periodic, synchronized oscillations.

Coupling of the circadian clock to the cell cycle may also be mediated by other regulatory mechanisms, e.g. inhibition of DNA transcription during mitosis. We will address the effect of such regulation in §2.3 below. A previous theoretical study [57], using the same model for the mammalian circadian clock [24], showed that the periodic inhibition of transcription can entrain the circadian clock. Forcing by the cell cycle in that study was implemented in a simplified manner, by representing transcriptional inhibition in the M phase by a square wave [57]. By contrast, the coupling of the circadian clock to the cell cycle is considered here explicitly, by linking detailed computational models for the two cellular networks.

### 2.2. Bidirectional coupling of the cell cycle and the circadian clock

Having shown that unidirectional coupling can lead either to entrainment of the cell cycle by the circadian clock or to entrainment of the circadian clock by the cell cycle, depending on the direction of coupling, the question arises as to what happens when the coupling becomes bidirectional? Can one oscillator still entrain the other or do the two oscillators synchronize at an intermediate period, or at some other period? To address this question we determined the dynamical behaviour of the coupled oscillators upon linking simultaneously the cell cycle to the circadian clock via the BMAL1-controlled rate of synthesis of *Wee1* mRNA, *v*_sw_, and the circadian clock to the cell cycle via the rate of phosphorylation of REV-ERBα by CDK1, *V*_Cdk1_, as schematized in electronic supplementary material, figure S1c. We first focus on the situation in which the autonomous period of the cell cycle (*T*_CC_) is fixed at the value of 20 h, prior to its coupling to the circadian clock, whose autonomous period (*T*_CR_) is fixed at the value of 24 h.

#### 2.2.1. Synchronization through bidirectional coupling: dependence on coupling strengths

**Figure 4.**
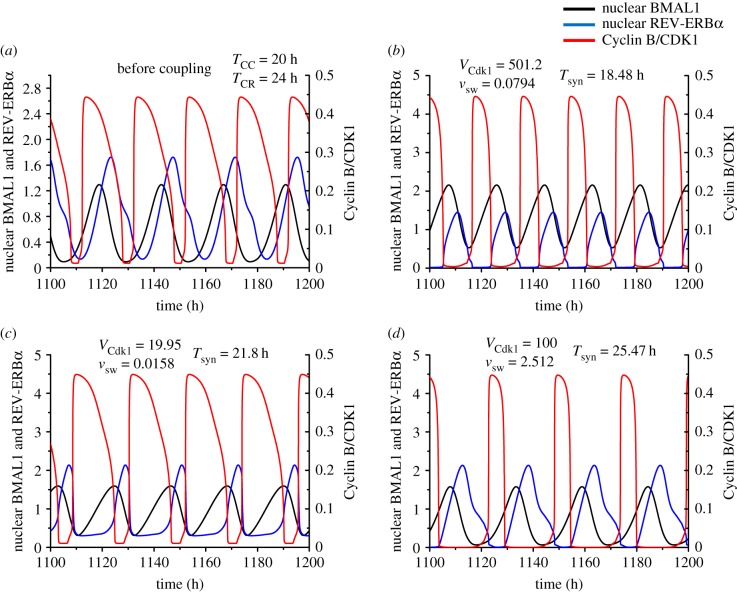
Bidirectional coupling of the circadian clock and the cell cycle, through BMAL1 induction of *Wee1* and REV-ERBα phosphorylation by CDK1. Before coupling, the cell cycle and the circadian clock oscillate independently with an autonomous period of 20 h and 24 h, respectively (*a*). Upon bidirectional coupling at *t* = 1110 h (vertical arrows), depending on the strengths of coupling, the cell cycle and the circadian clock can synchronize at a period shorter than 20 h (*b*), between 20 and 24 h (*c*), or longer than 24 h (*d*). In (*b*) where the synchronization period is 18.48 h, the coupling strength of the cell cycle to the circadian clock (*v*_sw_, in μMh^−1^) is 0.0794 and the reverse coupling strength (*V*_Cdk1_, in nMh^−1^) is 501.2. In (*c*) where the synchronization period is 21.8 h, *v*_sw_ = 0.0158 and *V*_Cdk1_ = 19.95. In (*d*) the synchronization period is 25.47 h, *v*_sw_ = 2.512 and *V*_Cdk1_ = 100. Curves in black, blue and red represent the time evolution of nuclear BMAL1, nuclear REV-ERBα and Cyclin B/CDK1, respectively. The transients in (*b*)–(*d*) last for one or two cycles. The data in (*b*), (*c*) and (*d*) correspond, respectively, to the points marked 1, 2 and 3 in figure 5*a*. (Online version in colour.)

In the absence of coupling, the two networks oscillate independently at their respective autonomous periods (figure 4*a*). As shown in figure 4*b*–*d* for three sets of values of the coupling strengths, the cell cycle and the circadian clock can readily synchronize in the presence of bidirectional coupling. However, in contrast to what occurs in the cases of unidirectional coupling considered in §2.2, we do not observe that one oscillator necessarily imposes its autonomous period on the other. Indeed, in figure 4*c*, bidirectional coupling of the circadian clock and cell cycle results in their synchronization at an intermediate period between 20 and 24 h. More unexpectedly we observed that the synchronization period, *T*_syn_, can be shorter than 20 h (figure 4*b*) or longer than 24 h (figure 4*d*), depending on the relative strengths of coupling measured by the BMAL1-controlled rate of synthesis of *Wee1* mRNA, *v*_sw_, and the CDK1-controlled rate of phosphorylation of REV-ERBα, *V*_Cdk1_. Thus, the two oscillators may synchronize at a period outside the range defined by their autonomous periods prior to coupling.

**Figure 5.**
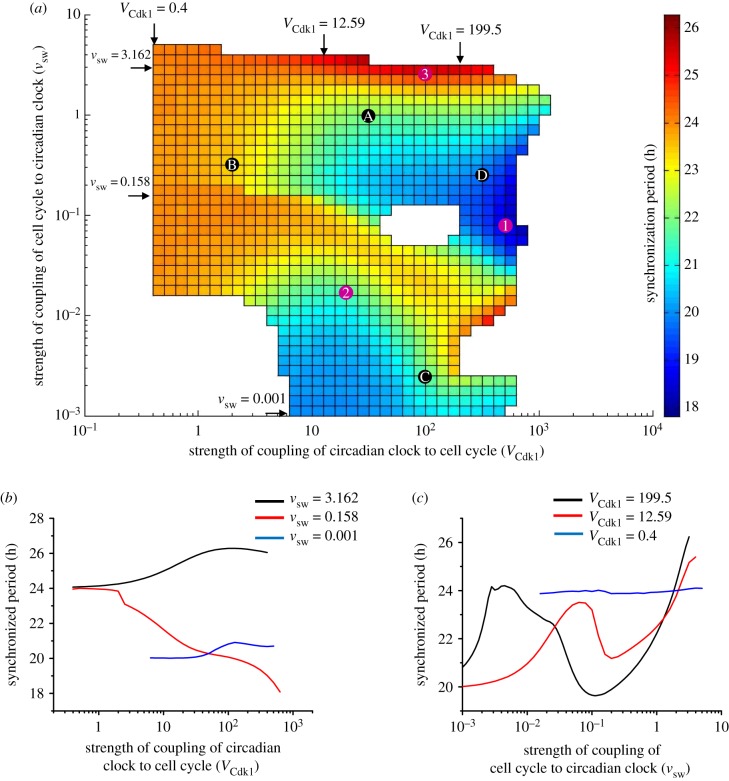
Bidirectional coupling: dependence of the synchronization period on the relative strengths of coupling of the cell cycle and the circadian clock. The cell cycle is coupled to the circadian clock through BMAL1 induction of *Wee1* while the circadian clock is linked to the cell cycle through REV-ERBα phosphorylation by CDK1. (*a*) Heat map showing how the period of synchronization varies as a function of the strength of coupling of the cell cycle to the circadian clock (*v*_sw_, in μMh^−1^) and of the strength of coupling of the circadian clock to the cell cycle (*V*_Cdk1_, in nMh^−1^). The diagram is established for an autonomous period *T*_CC_ = 20 h for the cell cycle and *T*_CR_ = 24 h for the circadian clock. Coloured regions indicate synchronization in the form of simple periodic oscillations; the colour code for the synchronization period, on the right, ranges from 17.8 to 26.3 h. Points marked 1, 2 and 3 refer to the synchronized oscillations shown in figure 4*b*, *c* and *d*, respectively. Points marked A, B, C and D correspond, respectively, to the situations considered in figure 6*a*–*d*. (*b*) Three horizontal sections through the heat map in (*a*) as a function of the strength of coupling of the circadian clock to the cell cycle, *V*_Cdk1_, at decreasing values of the strength of coupling of the cell cycle to the circadian clock, *v*_sw_. For *v*_sw_ = 3.162, or 0.158, as *V*_Cdk1_ increases, the period of synchronization ranges, respectively, from 24.1 h to 26.3 h (black curve) or from 24 h to 18.1 h (red curve), while it remains close to 20 h when *v*_sw_ = 0.001 (blue curve). (*c*) Three vertical sections through the heat map in (*a*) as a function of the strength of coupling of the cell cycle to the circadian clock, *v*_sw_, at decreasing values of the strength of coupling of the circadian clock to the cell cycle, *V*_Cdk1_. For *V*_Cdk1_ = 199.5 or 12.59, the synchronization period ranges from 19.65 to 26.23 h (black curve) or from 20 to 25.39 h (red curve) as *v*_sw_ increases, while it remains close to 24 h when *V*_Cdk1_ = 0.4 (blue curve). (Online version in colour.)

To extend these results we show in the two-dimensional graph in figure 5*a* how the synchronization period varies as a function of the two coupling strengths in the case where the autonomous cell cycle period is 20 h, while the circadian clock, as in all our simulations, has an autonomous period of 24 h. The diagram is presented in the form of a heat map (see the colour code on the right-hand side of the figure) where the synchronization period goes from a value less than 20 h (dark blue) to a value longer than 24 h (red); other regions on the map indicate periods between 20 and 24 h.

To quantify more precisely the dependence of the synchronization period on the coupling strengths in the case of bidirectional coupling we made three vertical and three horizontal sections through the two-dimensional diagram in figure 5*a*, at three increasing values of *v*_sw_ and *V*_Cdk1_, respectively. The results in figure 5*b* indicate that at low values of *v*_sw_ (blue curve), i.e. when the coupling of the cell cycle to the circadian clock is relatively weak compared with the strength of the reverse coupling, *T*_syn_ remains close to the cell cycle autonomous period *T*_CC_ of 20 h, regardless of the value of *V*_Cdk1_. Conversely, when the value of the coupling strength *V*_Cdk1_ of the circadian clock to the cell cycle is relatively low (figure 5*c*, blue curve) compared with the strength of coupling of the cell cycle to the circadian clock, *T*_syn_ remains close to the circadian period, regardless of the value of *v*_sw_.

However, as shown by the heat map in figure 5*a*, and by the black and red curves in figure 5*b*,*c*, when the two coupling strengths are of comparable magnitude the dependence of *T*_syn_ with respect to parameters *V*_Cdk1_ and *v*_sw_ becomes highly nonlinear and the synchronization period is less predictable. The numerical simulations further show that at high values of the coupling strength of the circadian clock to the cell cycle, *V*_Cdk1_ (figure 5*c*, black curve), the synchronization period increases from 21 to 24 h, then decreases to some 19.5 h before increasing again up to some 26 h, as the coupling strength of the cell cycle to the circadian clock, *v*_sw_, progressively increases. A similar profile for *T*_syn_ is also seen, with a reduced amplitude, for the curve in red in figure 5*c*. In figure 5*b*, values of *T*_syn_ longer than 24 h (black curve) or shorter than 20 h (red curve) are also observed.

What happens outside the coloured regions in the diagram in figure 5*a*? In electronic supplementary material, figure S2, based on the heat map of figure 5*a*, we selected points marked *a*–*h*, which all lie outside the coloured regions denoting synchronization of the cell cycle and the circadian clock in the form of simple periodic oscillations. We illustrate in the corresponding panels (*a*–*h*) of electronic supplementary material, figure S3 the dynamics of the bidirectionally coupled system in these points. At the bottom left in the diagram, both coupling strengths are very small so that the circadian clock and the cell cycle both oscillate independently at their autonomous period (electronic supplementary material, figure S3a). When the coupling between the two oscillators is weak but not negligible, quasi-independent oscillations occur with a minute modulation in the amplitude (electronic supplementary material, figure S3b). At points *c* and *d* in the white hole to the right of the centre of the diagram in electronic supplementary material, figure S2, synchronization occurs, not in the form of simple periodic oscillations as in the coloured parts of the diagram and illustrated in figure 4*b*–*d*, but in the form of period-2 oscillations, with two distinct peaks of each variable per period and two slightly distinct time intervals between the two successive peaks observed over a period (electronic supplementary material, figure S3*c*,*d*). These oscillations represent another mode of synchronization between the cell cycle and the circadian clock. Period-2 oscillations are also observed at point *e* in the white domain located in the upper right above the diagram in electronic supplementary material, figure S2, but the second peak in Cyclin B/CDK1 over a period has but a minute amplitude (electronic supplementary material, figure S3e).

Outside the diagram at the bottom right (point *f* in electronic supplementary material, figure S2) we observe synchronization in the form of period-3 oscillations, with three peaks of distinct amplitude per period (electronic supplementary material, figure S3f). Well above the coloured regions (point *g*) or well to the right (point *h*) one of the oscillators becomes silent while the other continues to cycle. This corresponds either to cell cycle arrest with a functional circadian clock (electronic supplementary material, figure S3 g), because of the high level of WEE1 which prevents cell proliferation, or to arrest of the circadian clock while the cell cycle continues to oscillate (electronic supplementary material, figure S3 h), when the levels of REV-ERBα and, hence, of BMAL1 fall outside the oscillatory range of the circadian clock mechanism when the activity of CDK1 becomes too large with respect to that of its inhibitor WEE1. Everywhere else in the coloured parts of figure 5*a* bidirectional coupling of the two oscillators results in their synchronization in the form of simple periodic oscillations characterized by a period indicated by the colour code. As will be stressed in the Discussion, cell cycle arrest may originate from a variety of causes, a major one being cell contact inhibition at high cell density [31,42].

In conditions of bidirectional coupling of the cell cycle and the circadian clock, each of the two oscillators acts as periodic input for the other. The range of coupling strengths, *V*_Cdk1_ and *v*_sw_, that allow synchronization can be estimated from the diagram in figure 5*a* for an autonomous cell cycle period of 20 h. The results indicate that the range of *V*_Cdk1_ values in which synchronization occurs varies according to the value of the other coupling strength, *v*_sw_ (see also figure 5*a*,*b*). Similarly, synchronization occurs in a range of *v*_sw_ values at a given value of *V*_Cdk1_ (figure 5*a*,*c*).

#### 2.2.2. Dependence of synchronization on autonomous periods of the cell cycle and the circadian clock

**Figure 6.**
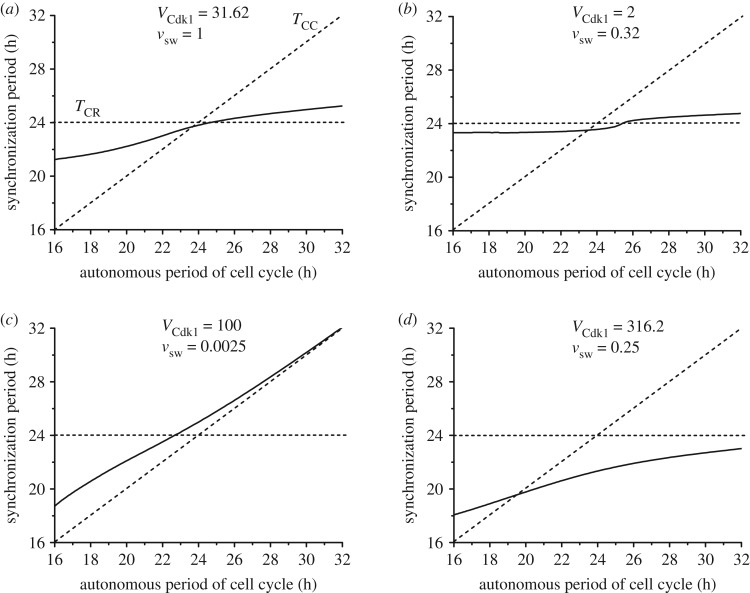
Bidirectional coupling: dependence of the synchronization period on the autonomous period of the cell cycle, *T*_CC_. As in figures 4 and 5, the cell cycle is coupled to the circadian clock through BMAL1 induction of *Wee1* while the circadian clock is linked to the cell cycle through REV-ERBα phosphorylation by CDK1. The autonomous period of the circadian clock *T*_CR_ is fixed at 24 h, while the autonomous period of the cell cycle increases from 16 to 32 h (by changing the scaling parameter *eps* from 26.9 to 13.4; see Section 1 in the electronic supplementary material). The values of the coupling strengths *V*_Cdk1_ (in nMh^−1^) and *v*_sw_ (in μMh^−1^) are of comparable magnitude in (*a*) (*V*_Cdk1_ = 31.62, *v*_sw_ = 1), while in (*b*) the coupling of the circadian clock to the cell cycle is weaker (*V*_Cdk1_ = 2, *v*_sw_ = 0.32). In (*c*) the coupling of the circadian clock to the cell cycle is much stronger than the reverse coupling (*V*_Cdk1_ = 100, *v*_sw_ = 0.0025), as in (*d*) where the curve was obtained for larger values of the two coupling strengths (*V*_Cdk1_ = 316.2, *v*_sw_ = 0.25). In each panel the synchronization period *T*_syn_ (solid line) increases gradually with *T*_CC_. The horizontal and diagonal dashed lines correspond, respectively, to *T*_syn_ = *T*_CR_ and *T*_syn_ = *T*_CC_. All synchronized oscillations are of the simple periodic type with one peak of each variable per period. The data in (*a*), (*b*), (*c*), and (*d*) correspond, respectively, to the points marked A, B, C and D in figure 5*a*.

The heat map in figure 5*a* was established for a cell cycle autonomous period *T*_CC_ of 20 h. To explore what happens at other values of *T*_CC_ we determined by numerical simulations the period of synchronization under bidirectional coupling over a range of values of *T*_CC_ extending from 16 to 32 h, with *T*_CR_ = 24 h. The results are shown in figure 6*a*–*d* for four pairs of values of the coupling strengths *v*_sw_ and *V*_Cdk1_ corresponding to points marked A–D in figure 5*a* (we recall that this diagram was established for *T*_CC_ = 20 h, while in figure 6 this is but one particular value of *T*_CC_, which now varies from 16 to 32 h). Figure 6*a* pertains to a situation in which the two coupling strengths are of comparable magnitude, while in figure 6*b* the coupling of the circadian clock to the cell cycle is weaker. In figure 6*c* the coupling of the circadian clock to the cell cycle is much stronger than the reverse coupling, as in figure 6*d*, where the curve has nevertheless been established for a larger coupling strength of the cell cycle to the circadian clock.

In each panel in figure 6 the horizontal dashed curve refers to the case where *T*_syn_ = *T*_CR_ = 24 h, while the diagonal dashed curve refers to *T*_syn_ = *T*_CC_. The two dashed lines define four regions in which the synchronization period *T*_syn_ is either (i) below *T*_CC_ and *T*_CR_ (right lower quadrant), between *T*_CC_ and *T*_CR_ with (ii) *T*_CC_ < *T*_syn_ < *T*_CR_ (left lower quadrant) or (iii) *T*_CR_ < *T*_syn_ < *T*_CC_ (right upper quadrant), or (iv) larger than both *T*_CC_ and *T*_CR_ (left upper quadrant).

We first observe that synchronization readily occurs in all cases considered in figure 6 over the full range of values of the cell cycle period. Second, in figure 6*a* the synchronization period is generally intermediate to the circadian and cell cycle autonomous periods; this corresponds to situations (ii) or (iii) as *T*_CC_ increases, except in a minute region near *T*_CC_ = 24 h, where *T*_syn_ is slightly shorter than 24 h. By contrast, in figure 6*c* the synchronization period is intermediate to *T*_CC_ and *T*_CR_ when *T*_CC_ is between 16 and 22.5 h, which corresponds to situation (ii); for *T*_CC_ values longer than 22.5 h, *T*_syn_ is above both *T*_CC_ and *T*_CR_—this corresponds to situation (iv)—and rises with *T*_CC_ until it approaches it when the latter exceeds 28 h. The situation in figure 6*b* is somewhat similar to that in figure 6*a*, but *T*_syn_ remains closer to *T*_CR_ = 24 h at all values of *T*_CC_ because the coupling of the cell cycle to the circadian clock is much stronger than the reverse coupling. Finally, in figure 6*d T*_syn_ goes from situation (ii) to (i) as *T*_CR_ rises, as *T*_syn_ begins to be below *T*_CR_ and closely above *T*_CC_ before becoming smaller than both *T*_CR_ and *T*_CC_.

**Figure 7.**
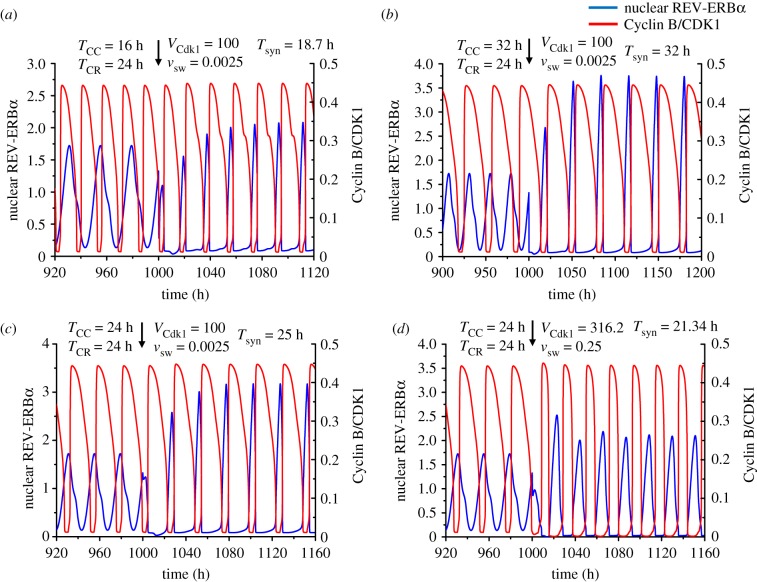
Bidirectional coupling of the circadian clock and the cell cycle for different values of the cell cycle autonomous period, *T*_CC_. As in figures 4–6, the cell cycle is coupled to the circadian clock through BMAL1 induction of *Wee1* while the circadian clock is linked to the cell cycle through REV-ERBα phosphorylation by CDK1. (*a*) Before coupling, the cell cycle and the circadian clock oscillate independently with an autonomous period of 16 h and 24 h, respectively. Upon coupling, with *V*_Cdk1_ = 100 (in nMh^−1^) and *v*_sw_ = 0.0025 (in μMh^−1^), the two oscillators synchronize at a period of 18.7 h. (*b*) When the autonomous period of the cell cycle is increased to 32 h, the cell cycle and the circadian clock synchronize after coupling at a period of 32 h. (*c*–*d*) When the autonomous period is 24 h for both the cell cycle and the circadian clock, the synchronized period upon coupling can be longer or shorter than 24 h, e.g. 25 h in (*c*) or 21.34 h in (*d*). The coupling strengths are *V*_Cdk1_ = 100, *v*_sw_ = 0.0025 in (*c*), and *V*_Cdk1_ = 316.2 and *v*_sw_ = 0.25 in (*d*). The scaling parameter *eps* is equal to 26.9 in (*a*), 13.4 in (*b*), and 17.9 in (*c*) and (*d*). (Online version in colour.)

Three examples of synchronized oscillations corresponding to figure 6*c* are shown in figure 7*a*–*c*, for *T*_CC_ = 16 h, 24 h and 32 h, respectively. In all cases synchronization occurs rapidly, in a few cycles. Interestingly, when both *T*_CC_ and *T*_CR_ are equal to 24 h in figure 7*c*, the synchronization period is close to 25 h. It should be noted that each oscillator is modified by incorporation of the coupling term that links it to the other; this in itself should impinge on the period of each of the two oscillators. Moreover, the bidirectional coupling of the cell cycle and the circadian clock produces a new dynamical system, consisting of a larger number of variables; the oscillatory behaviour of this extended system differs from that of each of its two components. In addition we show in figure 7*d* the oscillations for *T*_CC_ = *T*_CR_ = 24 h in the situation corresponding to figure 6*d*. The results in figure 7*c*,*d* show that, when both autonomous periods are equal to 24 h, the circadian clock and the cell cycle may synchronize at a period below or above 24 h.

Before coupling, the phase relationship between REV-ERBα and Cyclin B/CDK1 is variable, because the cell cycle and the circadian clock oscillate independently, with their own period. After bidirectional coupling starts, the oscillations become synchronized and the phase relationship between the two variables is fixed, with the maximum of Cyclin B/CDK1 corresponding to the trough of REV-ERBα (see, for example, figure 6*a*–*d*). This antiphase relationship reflects the experimentally based assumption that the circadian clock in the model is coupled to the cell cycle through Cyclin B/CDK1-catalysed phosphorylation of REV-ERBα, which leads to degradation of the protein.

**Figure 8.**
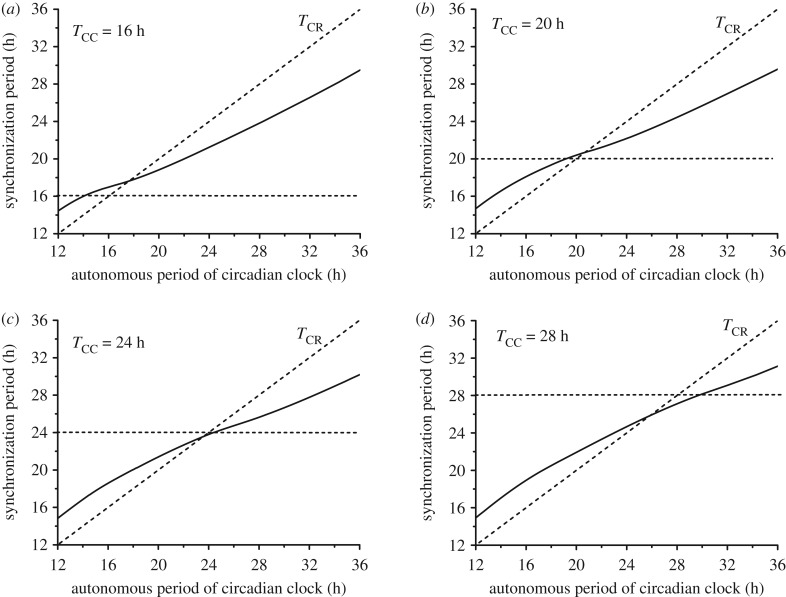
Bidirectional coupling: dependence of the synchronization period on the autonomous period of the circadian clock, *T*_CR_. As in figures 4–7, the cell cycle is coupled to the circadian clock via BMAL1 induction of *Wee1* while the circadian clock is linked to the cell cycle through REV-ERBα phosphorylation by CDK1 (see Sections 2 and 3 in the electronic supplementary material). The coupling strengths are fixed at the values *V*_Cdk1_ = 31.62 and *v*_sw_ = 1 considered in figure 6*a*. The autonomous period of the circadian clock increases from 12 to 36 h by progressively changing the scaling parameter *delta* from 2 to 2/3 (see Section 1 in the electronic supplementary material). As indicated in each panel, the cell cycle autonomous period *T*_CC_ is equal to 16 h (*a*), 20 h (*b*), 24 h (*c*) or 28 h (*d*). All synchronized oscillations are of the simple periodic type with one peak of each variable per period. Parameter values and initial conditions are listed in the electronic supplementary material.

The above results have been obtained upon varying the cell cycle autonomous period while holding the period of the circadian clock fixed at 24 h. Similar results are obtained when *T*_CR_ differs from 24 h, as observed, for example, in fibroblast cell cultures in which the circadian clock has a period of the order of 17 h [11,35,37]. As shown in figure 8 for four different values of the cell cycle autonomous period (*T*_CC_ = 16, 20, 24 or 28 h), synchronization readily occurs when the autonomous period of the circadian clock *T*_CR_ varies from 12 to 36 h. As in figure 6 established as a function of *T*_CC_, the synchronization period is generally between the autonomous periods of the cell cycle and the circadian clock.

### 2.3. Multiple modes of bidirectional coupling

As recalled in §2.1, the cell cycle is controlled by the circadian clock though multiple modes of coupling. Thus, BMAL1 not only induces the kinase WEE1 [32], but also directly or indirectly regulates the levels of other cell cycle proteins, including Cyclin E [33,34], Cyclin B [35] and the CDK inhibitor p21 [36]. We previously showed that unidirectional coupling of the cell cycle to the circadian clock, leading to entrainment of the cell cycle to 24 or 48 h, also occurs when BMAL1 induces one, two or all of the proteins WEE1, Cyclin E and p21 [56].

#### 2.3.1. Multiple modes of coupling the cell cycle to the circadian clock

Do multiple modes of coupling of the cell cycle to the circadian clock affect their synchronization in the case of bidirectional coupling? To address this question, along with the phosphorylation of REV-ERBα by CDK1 we included in the coupled system the indirect negative regulation of Cyclin E by BMAL1, which inhibits c-Myc, an inducer of Cyclin E [33,34]. This negative regulation of Cyclin E by BMAL1 will be incorporated, as described in Section 4 in the electronic supplementary material, first without, and then in conjunction with, BMAL1 induction of *Wee1*. The results in electronic supplementary material, figure S4 indicate that synchronization can occur when Cyclin E, instead of WEE1 (electronic supplementary material, figure S4a,b), or together with WEE1 (electronic supplementary material, figure S4c,d), is regulated by BMAL1. As shown in electronic supplementary material, figure S5a,b, when both WEE1 and Cyclin E are controlled by BMAL1, the two effects may combine to transform the synchronized period-2 or period-3 oscillations represented in electronic supplementary material, figure S2c,f into simple periodic oscillations. The multiplicity of the modes of cell cycle coupling to the circadian clock can thus contribute to robust synchronization of the two cellular rhythms in conditions of bidirectional coupling.

#### 2.3.2. Multiple modes of coupling the circadian clock to the cell cycle

As to coupling the circadian clock to the cell cycle, besides the regulation of REV-ERBα by CDK1 considered above, another mode of coupling may result from the inhibition of transcription at mitosis [58,59], as noted by Bieler *et al*. [38]. Earlier experimental studies established the role of CDK1 in the inhibition of the transcription machinery through phosphorylation of some of its components [58,59]. To take into account this regulation in a phenomenological manner, we multiplied in the model for the circadian clock all terms representing synthesis of mRNA by the term $K_{\mathrm{Icdk}1}^{\mathrm{ncdk}1}/(K_{\mathrm{Icdk}1}^{\mathrm{ncdk}1}+Mb^{\mathrm{ncdk}1}),$ which takes the form of an inhibitory Hill function where *Mb* represents the concentration of Cyclin B/CDK1, *K*_Icdk1_ is an inhibition constant and *ncdk1* is the degree of cooperativity of the inhibition process (see Section 5 in the electronic supplementary material). As shown in electronic supplementary material, figure S6, this regulation of the circadian clock by the cell cycle is capable of eliciting by itself the entrainment of the circadian clock, the period of which shifts to that of the cell cycle, both when *T*_CC_ is shorter or longer than *T*_CR_. Such a result extends those of previous simulations [57] in which the same model for the circadian clock was shown to be entrained by the cell cycle through periodic inhibition of transcription in M phase, represented by a square wave. Here, by contrast, the cell cycle is represented explicitly by a detailed model for the CDK network.

#### 2.3.3. Coexistence of multiple modes of synchronization: birhythmicity and trirhythmicity

**Figure 9.**
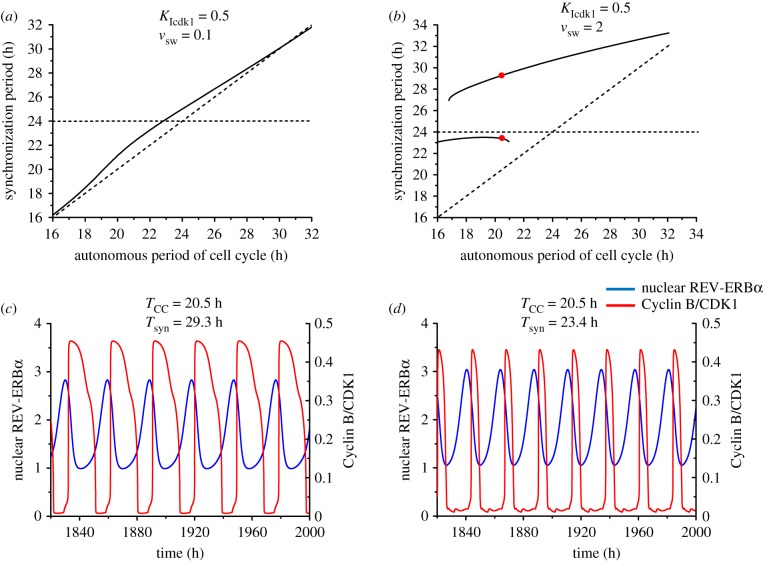
Bidirectional coupling via mitotic repression of transcription, controlled by CDK1, and BMAL1 induction of *Wee1*: the synchronization period as a function of the autonomous period of the cell cycle, and birhythmicity. The autonomous period of the circadian clock, *T*_CR_, is fixed at 24 h, while the autonomous period of the cell cycle, *T*_CC_, increases from 16 to 32 h (by changing the scaling parameter *eps* from 26.9 to 13.4; see Section 1 in the electronic supplementary material). (*a*) When *K*_Icdk1_ = 0.5 µM and *v*_sw_ = 0.1 µMh^−1^, the synchronization period increases gradually from 16 to 32 h, and the synchronization period is close to the period of the cell cycle. (*b*) When *K*_Icdk1_ = 0.5 and *v*_sw_ = 2, the synchronization period is longer than the autonomous periods of the cell cycle and the circadian clock. Two stable modes of synchronization (birhythmicity) coexist in a range of *T*_CC_ values extending from 16.8 to 21 h. (*c*,*d*) The two coexisting modes of synchronization in the domain of birhythmicity for *T*_CC_ = 20.5 h, corresponding to the red dots in (*b*). The bidirectionally coupled system can synchronize at a period of 29.3 h (*c*) or 23.4 h (*d*), depending on the initial conditions (listed in Section 9 in the electronic supplementary material). (Online version in colour.)

We now combine the BMAL1 induction of *Wee1* with the reverse coupling of the circadian clock to the cell cycle via mitotic repression of transcription, controlled by CDK1, rather than REV-ERBα phosphorylation by CDK1. This situation represents another realization of bidirectional coupling. We show in figure 9*a* that synchronization again occurs over the whole range of *T*_CC_ values investigated, i.e. from 16 to 32 h. Because repression by CDK1 of transcription directly impinges on many variables in the circadian clock model, the coupling of the circadian clock to the cell cycle is stronger, so that *T*_syn_ is always close to *T*_CC_.

To determine what happens if we increase the strength of reverse coupling, we increased the value of *v*_sw_ in figure 9*b*. Again synchronization occurs, further away from the cell cycle period, but a new phenomenon is observed. Indeed, in a range of *T*_CC_ values between 16.5 and 21 h in the case considered, two branches of *T*_syn_ overlap: in this range, the circadian clock and the cell cycle can synchronize in two different ways, corresponding to two distinct periods of synchronization. We illustrate in figure 9*c*,*d* the coexistence between the two coexisting modes of synchronization, corresponding to the red dots in figure 9*b*, for the value of *T*_CC_ = 20.5 h. For this value of *T*_CC_ the two oscillators can synchronize at a period of 29.3 h (figure 9*c*) or 23.4 h (figure 9*d*), depending on initial conditions. As illustrated in figure 9*c*,*d*, the two coexisting synchronized oscillations differ not only by the period but also by the waveform and amplitude. This phenomenon, referred to as birhythmicity [60], has been observed in a variety of biochemical models [61–64] in which it originates from the interplay between several oscillatory mechanisms.

The results in figure 9 were obtained when the degree of cooperativity *ncdk1* of the inhibition by CDK1 of mRNA synthesis is equal to 2. In the absence of cooperativity (*ncdk1* = 1), the bidirectional coupling can also lead to synchronization and a similar diagram for *T*_syn_ as a function of *T*_CC_ is obtained (electronic supplementary material, figure S7a). It includes a region of birhythmicity, but also a narrow domain of trirhythmicity in which the circadian clock and the cell cycle can synchronize in three different ways corresponding to three distinct types of simple periodic oscillations, which coexist in the same conditions. The three modes of synchronized oscillations shown in electronic supplementary material, figure S7b–d correspond to the red dots for the value *T*_CC_ = 20.5 h in electronic supplementary material, figure S7a. These three regimes of synchronized oscillations, each of which differs by the amplitude and by the period (*T*_syn_ = 23.74, 25.2 or 30.89 h, in the case considered), can be reached when starting from different initial conditions.

**Figure 10.**
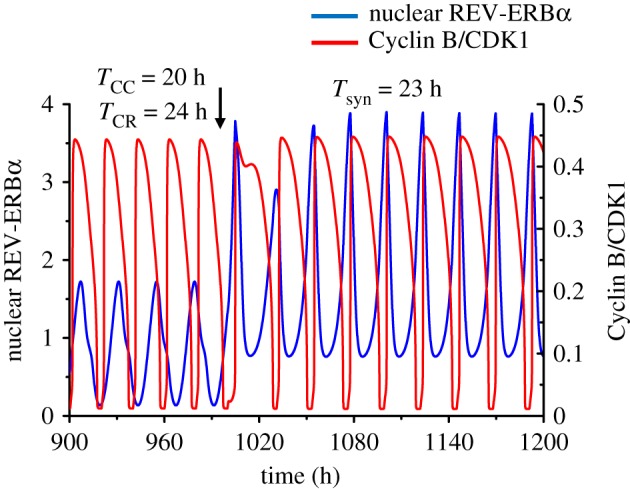
Synchronization readily occurs when two modes of coupling of the cell cycle to the circadian clock and two modes of coupling of the circadian clock to the cell cycle are considered simultaneously. The cell cycle and the circadian clock respectively have a period of *T*_CC_ = 20 h and *T*_CR_ = 24 h before coupling. Upon bidirectional coupling, the synchronized circadian clock and the cell cycle oscillate at a period of *T*_syn_=23 h. The circadian clock is coupled to the cell cycle via mitotic repression of transcription and REV-ERBα phosphorylation, both under the control of CDK1. The cell cycle is coupled to the circadian clock via BMAL1 induction of *Wee1* and negative regulation of Cyclin E by BMAL1. The coupling strengths are *K*_Icdk1_ = 0.5 µM, *v*_sw_ = 0.1 µMh^−1^, *V*_Cdk1_ = 3.16 nMh^−1^ and *v*_sce_ = 0.005 µMh^−1^. (Online version in colour.)

Finally, synchronization readily occurs when two modes of coupling of the cell cycle to the circadian clock—namely, regulation by BMAL1 of both WEE1 and Cyclin E—together with two modes of coupling of the circadian clock to the cell cycle—i.e. phosphorylation of REV-ERBα by CDK1 and CDK1-controlled inhibition of mRNA synthesis—are considered simultaneously. In the case illustrated in figure 10, which incorporates these four modes of coupling, a cell cycle of 20 h period is shown to readily synchronize with the circadian clock (*T*_CR_ = 24 h) at the intermediate period of 23 h.

#### 2.3.4. For some couplings, synchronization may occur at very long periods

So far we considered that the circadian clock is controlled by the cell cycle in two possible ways, via the enhanced degradation of REV-ERBα through phosphorylation by CDK1—which leads to increased expression of *Bmal1*—and/or the inhibition of a component of the transcription machinery through phosphorylation by CDK1. The latter enzyme, CDK1, or other CDKs, may well have additional effects on the cell cycle, which remain to be discovered. Thus, CDK5, a cyclin-dependent kinase active in neuronal processes such as neurogenesis and axon guidance, was found to increase the rate of CLOCK degradation through direct phosphorylation of the protein [65]. To test the effect of such negative regulation of a clock component by a CDK, we investigated the effect of a putative inhibition of BMAL1 (which rapidly forms a complex with CLOCK in the circadian clock model) through phosphorylation by CDK1, even though no experimental evidence supports this mode of regulation.

**Figure 11.**
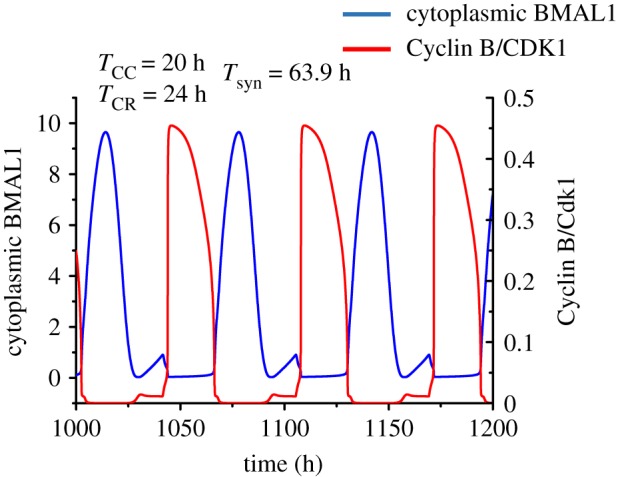
Synchronization at very long periods, when bidirectional coupling of the circadian clock and the cell cycle occur through BMAL1 induction of *Wee1* and cytoplasmic BMAL1 phosphorylation by CDK1. While the autonomous periods of the cell cycle and the circadian clock are 20 h and 24 h, respectively, upon bidirectional coupling the cell cycle and the circadian clock synchronize at a period of 63.9 h. The curves showing the time evolution of Cyclin B/CDK1 and cytoplasmic BMAL1 are obtained as described in Section 7 in the electronic supplementary material, for *v*_sw_ = 0.3 µM h^−1^ and *V*′_Cdk1_ = 180 nM h^−1^. (Online version in colour.)

The results indicate that when bidirectional coupling between the cell cycle and the circadian clock incorporates this negative regulation instead of the phosphorylation of REV-ERBα by CDK1, a new phenomenon may be observed. While synchronization again occurs in general at periods between the autonomous periods of the cell cycle (20 h) and the circadian clock (24 h), sometimes the synchronization period is much longer and can reach values up to 63.9 h, as illustrated in figure 11. What is the mechanism underlying such a phenomenon? We believe that it originates from the mutual inhibition of the cell cycle by the circadian clock (via induction by BMAL1 of the CDK1 inhibitor WEE1) and of the circadian clock by the cell cycle (via the putative inhibition of BMAL1 by CDK1). In the course of oscillations, the rise in BMAL1 coincides with a decrease in CDK1 (figure 11), because the two systems oscillate in antiphase. After a full-amplitude peak in BMAL1, the next peak is aborted, however, by the delayed rise in CDK1. As a result, one full-amplitude peak out of two is skipped for both BMAL1 and CDK1: the cell cycle and the circadian clock still synchronize, but the period of synchronization is greatly enlarged.

Even if negative regulation of BMAL1 by CDK1 is not supported by experimental observations, contrary to the control of REV-ERBa through CDK1, which enhances BMAL1, the results of figure 11 indicate that unusual, more counterintuitive modes of synchronization in principle become possible when considering other types of coupling between the two regulatory networks.

## 3. Discussion

Experimental observations in unicellular [1–5] and multicellular organisms [6–9], including mammals [11–13,16,17,32–39], have established the existence of interactions between the cell cycle and the circadian clock. In mammalian cells, BMAL1, a key transcription factor at the core of the circadian clock network, directly or indirectly controls the expression of several cell cycle genes such as those coding for WEE1, p21, Cyclin E or Cyclin B [32–36]. These regulations allow for unidirectional control of the cell cycle by the circadian clock. There is also evidence for control of the circadian clock by the cell cycle [37,38]. Although the molecular bases for such reverse control remain to be explored in further detail, a first mode of regulation was recently characterized, by which the Cyclin-dependent kinase CDK1, which controls the G2/M transition in the cell cycle, controls phosphorylation of the circadian protein REV-ERBα and thereby marks it for degradation [39]. A second mode by which the cell cycle may influence the circadian clock stems from the inhibition of transcription at mitosis [58,59]. These regulations indicate that the coupling of the cell cycle and the circadian clock is bidirectional.

To assess the effect of the unidirectional coupling of the cell cycle to the circadian clock we previously coupled, via BMAL1, the detailed computational model proposed for oscillations in the CDK network driving the mammalian cell cycle [25] to a detailed computational model for the mammalian circadian clock [24]. We showed that such unidirectional coupling through BMAL1 involving the induction of *Wee1* or *p21* and/or the repression of *Cyclin E* can lead to entrainment of the cell cycle by the circadian clock [56]. Depending on its autonomous period *T*_CC_ prior to coupling, the cell cycle can be entrained to oscillate at a period of 24 or 48 h (see also figure 2, which illustrates entrainment of the cell cycle by the circadian clock through unidirectional coupling for a value of *T*_CC_ below or above 24 h). Outside the domains of entrainment, the system evolves to a stable steady state or complex oscillations occur [56]. Within the domains of entrainment, the coexistence between different modes of entrainment to 24 or 48 h can sometimes be observed [64]. A number of other studies have examined theoretically the consequences of coupling the cell cycle unidirectionally to the circadian clock [66–68].

To address the effect of the reverse unidirectional coupling, we took into account the control exerted by the cell cycle on the circadian clock through phosphorylation of REV-ERBα by Cyclin B/CDK1 [39]. We showed in figure 3 that this regulation, considered alone, can by itself support the entrainment of the circadian clock by the cell cycle.

In the case of unidirectional coupling, one of the oscillators is driving the other and imposes its period, or a multiple of its period. Thus, when the cell cycle is driven by the circadian clock, it adopts a period of 24 or 48 h, depending on the value of *T*_CC_ [56]. Likewise, when the circadian clock is driven by the cell cycle, the two oscillators synchronize at the cell cycle period, as the circadian period *T*_CR_ goes from 24 h to the value of *T*_CC_ = 20 h or 28 h in the case considered in figure 3.

The main goal of this work was to determine what happens when the coupling becomes bidirectional: can the circadian clock and the cell cycle synchronize, and, if so, at what period? We addressed the effect of bidirectional coupling by incorporating simultaneously the control exerted by the circadian transcription factor BMAL1 on the cell cycle via its induction of *Wee1*, and the control exerted by the cell cycle on the circadian clock via phosphorylation of REV-ERBα by Cyclin B/CDK1. Numerical simulations show that such bidirectional coupling of the cell cycle and the circadian clock results in the robust synchronization of the two cellular rhythms. Synchronization readily occurs in the form of simple periodic oscillations in a large domain of coupling strengths (figures 4–8).

In contrast to the situation of unidirectional coupling—except in extreme conditions, when one of the couplings becomes negligible (see figure 5*a*, and blue curves in figure 5*b*,*c*)—none of the oscillators imposes its period on the other. Depending on the relative coupling strengths, the two oscillators may either synchronize at a period intermediate between their autonomous periods *T*_CC_ and *T*_CR_ prior to coupling or, somewhat more surprisingly, at a period below or above the range defined by the autonomous periods (figures 4–8). Even when the autonomous periods of the cell cycle and circadian clock are both equal to 24 h, their bidirectional coupling may lead to their synchronization at a period above (figure 7*b*) or below (figure 7*d*) 24 h. This counterintuitive prediction based on numerical simulations of the comprehensive model for the coupled cell cycle and circadian networks represents an unexpected consequence of bidirectional coupling of the two cellular rhythms.

To comprehend how the synchronization period may be shorter or longer than the autonomous period of either the cell cycle or the circadian clock, two factors must be taken into account. First, the presence of an additional coupling term by itself modifies the dynamical properties, including the period, of each oscillator with respect to the situation prior to coupling. Second, the coupled circadian–cell cycle system represents a novel, more complex oscillatory system containing a larger number of variables as well as the additional regulations corresponding to bidirectional coupling. The period of oscillations of the bidirectionally coupled system may differ, sometimes by several hours, as shown here, from the autonomous periods of the two oscillators that constitute it. Nevertheless, the observation that the synchronization period can lie outside the range defined by the two autonomous periods is counterintuitive. In future work we plan to investigate in further detail this phenomenon, as well as other patterns of synchronization, including multi-rhythmicity, in a coupled system composed of reduced models for the circadian clock [40,41] and for the cell cycle [54,69]. The present results on the different patterns of synchronization in complex, realistic models paves the way for a detailed analysis of synchronization patterns in simplified models for the two cellular rhythms.

Even more counterintuitive is the finding of extremely long periods of synchronization when the circadian clock and the cell cycle are controlled through mutual inhibition (figure 11). However, in the absence of experimental evidence for a negative control of BMAL1 by CDK1, such a situation remains putative. The prediction that the two systems may sometimes synchronize at very long periods nevertheless extends the range at which synchronization may occur.

Bidirectional coupling results in the robust synchronization of the cell cycle and the circadian clock. Over several orders of magnitude of the two coupling strengths (figure 5*a*), bidirectional coupling produces simple periodic oscillations of the cell cycle and the circadian clock with a synchronization period in the range 18–26 h when coupling a cell cycle of 20 h to the circadian clock of 24 h. Similar results are obtained (figure 8) when *T*_CR_ differs from 24 h, as observed, for example, in fibroblast cell cultures in which the circadian clock has a period of the order of 17 h [10,34,36]. In a few regions of the diagram of figure 5*a*, synchronization takes the form of period-2 (electronic supplementary material, figure S2c–e) or period-3 oscillations (electronic supplementary material, figure S2f). Outside the domain of synchronization in figure 5*a*, depending on the relative values of WEE1 and CDK1, either the cell cycle is arrested while the circadian clock continues to tick (electronic supplementary material, figure S2 g) or the cell cycle oscillates while the circadian rhythm stops (electronic supplementary material, figure S2 h). Altering the level of BMAL1, through the control of REV-ERBα by CDK1, can indeed bring the circadian clock to a halt [26]. On the other hand, cell cycle arrest may be due to a change in parameters internal to the CDK network [31,42], or to contact inhibition of cell proliferation at high cell density, which is mediated by cadherin adhesion molecules via the Hippo/YAP signalling pathway [31], as occurs when cells reach confluence.

The fact that the synchronization period depends in a non-monotonous manner on the coupling strengths in figure 5*b*,*c* is reminiscent of the non-monotonous variation of the period as a function of parameters in models for the mammalian clock [24,26,70,71]. Another mode of synchronization may result from intercellular coupling, as shown for the circadian clock in cells that initially oscillate with slightly different periods. Thus the synchronization period was shown to depend on the strength of intercellular coupling in a multicellular model for the circadian clock in suprachiasmatic nuclei (SCN) [72]. In the present study, we considered other cell types, which undergo proliferation, and focused on the coupling, within each cell, of two distinct oscillators that control, respectively, the cell cycle and the circadian clock.

In the case of unidirectional coupling of the cell cycle to the circadian clock, we previously showed that outside the domains of entrainment to 24 or 48 h complex periodic or aperiodic oscillations may occur [56]. The physiological significance or the possible pathological consequences of such complex oscillations in the cell cycle remain an open question. By contrast, in the case of bidirectional coupling, no evidence for chaos or complex periodic oscillations was obtained—besides period-2 or period-3 oscillations—even though more complex behaviour cannot be ruled out. A large number of numerical simulations indicate that bidirectional coupling results in synchronization in the form of simple periodic oscillations (see, for example, figures 4, 7 and 10). Such a pattern of robust synchronization is observed over a large range of values of the two coupling strengths and of the autonomous period of the cell cycle prior to coupling (figure 6). Bidirectional coupling therefore appears to stabilize simple periodic oscillations in the circadian clock and cell cycle networks. When the two oscillators are coupled bidirectionally, at sufficiently large coupling strengths they become interlocked and oscillate ‘hand in hand’. A major effect of bidirectional coupling is therefore to enhance the robustness of synchronized oscillations and to reduce the likelihood of complex oscillatory behaviour that could be detrimental to both the cell cycle and the circadian clock. The addition of a single link from the cell cycle to the circadian clock transforms their coupling from unidirectional to bidirectional. While this modification of the evolution equations may look deceptively minor, it represents a major change because it has profound consequences for the synchronization and stabilization of the coupled cell cycle–circadian clock system.

In studying the effect of bidirectional coupling of the cell cycle and the circadian clock we initially focused on one mode of coupling in each direction; namely, BMAL1 induction of *Wee1*, on one hand, and, on the other hand, phosphorylation of REV-ERBα by CDK1. Other modes of coupling the cell cycle to the circadian clock have been uncovered, such as the direct or indirect control by BMAL1 of the levels of Cyclin E, Cyclin B and p21. Likewise, additional modes of coupling the circadian clock to the cell cycle begin to be characterized. Thus the process of DNA transcription impinges on the operation of the circadian clock [58,59,73], and this may in turn lead to entrainment of the latter by the cell cycle, as shown in §2.3 and electronic supplementary material, figure S5, which extend the results of previous modelling studies [57].

To address the effects of multiple modes of bidirectional coupling on synchronization, we first considered the effect of dual control, respectively positive and negative, of WEE1 and Cyclin E by BMAL1, in conditions where the reverse coupling occurs through phosphorylation of REV-ERBα by CDK1. The results indicate that synchronization of the cell cycle and the circadian clock also occurs in such conditions, much as when BMAL1 control of the cell cycle only occurs via WEE1 (electronic supplementary material, figure S3). Robustness of synchronization appears to be enhanced by dual control through BMAL1, compared with the situation where only one mode of coupling the cell cycle to the circadian clock is considered (electronic supplementary material, figure S4).

On the other hand, we considered the dual regulation by CDK1 of the circadian clock through (i) REV-ERBα phosphorylation [38] and (ii) inhibition of transcription by RNA polymerase [58,59], in the presence of *Wee1* induction by BMAL1 [32]. Here also, the two modes of control of the circadian clock by the cell cycle, alone or together, can elicit the synchronization of the two cellular rhythms. In some instances of such bidirectional coupling, numerical simulations revealed that the two oscillators may synchronize in two or three ways corresponding to the coexistence of two (figure 9) or three (electronic supplementary material, figure S6) different types of stable oscillations, characterized by distinct waveforms and periods. These results corroborate and extend those that we recently reported in the case of unidirectional coupling of the cell cycle to the circadian clock [64]. In that case, however, the coexisting oscillations correspond to distinct modes of entrainment of the cell cycle to a period of 24 or 48 h, or to complex oscillations of a period of 96 h, which is a multiple of 24 h. Here, by contrast, multi-rhythmicity in figure 9 or electronic supplementary material, figure S6 corresponds to different modes of synchronization in the form of simple periodic oscillations, with periods distinct from the autonomous periods of either the circadian clock or the cell cycle. This theoretical prediction is, again, counterintuitive, and cannot be readily explained in terms of a simple biophysical mechanism. Such multi-rhythmicity represents a property of nonlinear oscillators, which is brought to light by computational modelling. We hope that the present results on multi-rhythmicity, together with those reported in an earlier publication [64], will contribute to stimulating an experimental investigation of the phenomenon.

In a cell population such multi-rhythmicity could lead to cellular heterogeneity, with respect to circadian and cell cycle dynamics, much as the coexistence of a stable steady state and a stable periodic regime is a source of heterogeneity in modelling the cell cycle dynamics in a cell population [54,74]. If multiple modes of synchronization coexist within a given cell population, some cells might thereby gain a proliferative advantage if the duration of their cell cycle is reduced. The question arises as to whether such multi-rhythmicity might be involved in the occurrence of three peaks of cell division observed over 24 h in a population of NIH3T3 fibroblasts (see fig. 6E in [11], and a related observation in fig. 6 in [37]). Interestingly, such multiple peaks were also observed in a model of a cell population in which the cell cycle is described by a stochastic automaton entrained by the circadian clock with cell cycle phase durations randomly distributed around mean values (see fig. 4E in [75], and also [37]).

The effects of bidirectional coupling were addressed in greatest detail for the case where the cell cycle and the circadian clock are linked through a single mode of coupling in each direction (figures 4–9). Synchronization also occurs when two modes of coupling in one direction combine with one mode of coupling in the other direction (electronic supplementary material, figure S3). Considering all couplings together yields similar results. Thus, the simultaneous activation of the dual effect of BMAL1 on the cell cycle and of the dual regulation of the circadian clock by CDK1 readily leads to synchronization of the cell cycle and the circadian clock (figure 10).

If the cell cycle and the circadian clock can robustly synchronize through bidirectional coupling, it remains to be seen whether or to what extent such coupling occurs in physiological conditions. First, it is important to stress that our analysis pertains to the situation in which the cell cycle behaves as a self-sustained oscillator, which corresponds to conditions of cell proliferation. While this may be true in early development, in isolated cells in rich media or in growing tumours, in many tissues most cell are resting [76,77]. Then the cell cycle operates in a stable state corresponding to cell cycle arrest. What happens with bidirectional coupling between the circadian clock and the cell cycle when the latter is arrested? Besides the effect of cell density, cells may stop proliferating because of a lack of sufficient amounts of growth factors in the medium, or because of a change in some parameter that would bring the CDK network into a stable steady state. In electronic supplementary material, figure S8, we illustrate the effect of bidirectional coupling when the cell cycle is arrested because the level of growth factor is too low (electronic supplementary material, figure S8a) or because the level of CDC25 is too high (electronic supplementary material, figure S8b). In these conditions the cell cycle stops with a low or a high level of CDK1 activity, respectively. In both cases the cell cycle remains arrested as long as its coupling to the circadian clock is weak, although CDK oscillations may in principle resume when the coupling becomes sufficiently large. Moreover, the circadian clock period of 24 h slightly changes to 24.4 h (electronic supplementary material, figure S8a) or 24.24 h (electronic supplementary material, figure S8b), owing to the action of Cyclin B/CDK1, which promotes degradation of REV-ERBα and thereby affects the level of BMAL1.

In considering the physiological significance of our results, the question arises as to the effect of the light–dark (LD) cycle on the bidirectional coupling between the circadian clock and the cell cycle. In mammals the LD cycle primarily affects cells of the SCN in the hypothalamus, which behave as a circadian pacemaker [20–22]. In physiological conditions, peripheral circadian clocks in other tissues [21,22] are influenced indirectly by light acting on the SCN, via signals present in the serum, such as glucocorticoids [21,22,78]. Besides light, circadian variations in body temperature may also act as a zeitgeber [79]. *In vivo*, various cell cycle genes are expressed in a circadian manner in human tissues such as oral mucosa, skin, bone marrow or liver [16–18,32]. By contrast, cells in cultures are not subjected to forcing by the LD cycle. While cell cultures do not represent a fully physiological situation, they provide a most useful tool to gain insights into the molecular links between the cell cycle and the circadian clock in mammalian cells [11,35–38,80].

To extend the above results, which directly correspond to the situation encountered in cell cultures, we performed simulations of bidirectional coupling of the cell cycle (CC) to the circadian clock (CR) in the presence of forcing of the circadian clock by the LD cycle. In the model the effect of light is to enhance the expression of the *Per* gene [24]. The effect of the LD cycle depends on the relative strengths of coupling of CR to LD and CC, and of CC to CR. Three domains are found, as follows. (i) When the strength of coupling of CR to LD is relatively weak, synchronization of the cell cycle and the circadian clock occurs as in the absence of forcing by LD. (ii) When the strength of coupling of CR to LD is stronger than the coupling of CR to CC, the cell cycle and the circadian clock synchronize with LD at a period of 24 h. (iii) At intermediate strengths of coupling of CR to LD, more or less complex oscillations occur for the circadian clock and, to a lesser degree, for the cell cycle. These results, summarized in electronic supplementary material, figure S9, suggest that, if the coupling of the circadian clock to the LD cycle is stronger than its coupling to the cell cycle, the latter may not be able to synchronize with the circadian clock at an intermediate, non-circadian period. Whether the cell cycle is then entrained by the circadian clock and, indirectly, by the LD cycle will depend on the strength of coupling of the cell cycle to the circadian clock.

In proliferating cells, in the absence of coupling to the LD cycle, we explored the dynamics of the bidirectionally coupled system over a large range of values of the various coupling strengths, because the magnitude of the coupling strengths, which remains to be determined, may vary according to cell type and developmental conditions [8]. The spectrum of dynamical behaviour of the coupled cell cycle–circadian clock system is wide: it ranges from (i) quasi-independent oscillations, when the two coupling strengths are weak, to (ii) quasi-unidirectional coupling, when one coupling strength predominates over the other, which leads to synchronization in the form of entrainment of the strongly coupled oscillator by the dominant one which imposes its period, or (iii) robust synchronization of the cell cycle and the circadian clock when the two coupling strengths are of comparable magnitude. Thus, depending on the relative strengths of coupling of the two oscillators, the dynamics of the coupled system may range from independent oscillations to entrainment by a dominant oscillator (cell cycle or circadian clock), or synchronization of the two oscillators at a period that may be intermediate between the autonomous period of the cell cycle and the circadian clock prior to coupling, or outside the range defined by these autonomous periods. Which of these modes of dynamic behaviour is selected may vary in different cell types, and in a given type according to the conditions encountered by the cell [8,38].

Insights into the links between the cell cycle and the circadian clock can in principle be gained from studying the dependence of the two processes on temperature. The fact that the circadian clock maintains temperature compensation, in contrast to the cell cycle, in cultured NIH3T3 fibroblasts [81] suggests that temperature compensation, which is a hallmark of circadian rhythms, arises from compensatory mechanisms specific to circadian clocks, which are not transmitted to the cell cycle. Either the cell cycle is uncoupled from the circadian clock in these cells or the effect of temperature is stronger than any unidirectional or bidirectional coupling. Alternatively, synchronization may break down when the two autonomous periods become too far apart or when the coupling strengths change as the temperature increases.

Given the role of REV-ERBα in the putative coupling of the circadian clock to the cell cycle, it would be interesting to determine the duration of the cell cycle in fibroblast cultures for the case of REV-ERBα knockout mice. In these mice the difference in autonomous period for the circadian clock changes by about 0.4–0.6 h, as it goes roughly from about 23.8 h to 23.4 h in continuous darkness, and from 24.6 h to 24.0 h in continuous light when comparing wild-type with REV-ERBα knockout mice [82]. The model predicts that a slight decrease in the rate of *Rev-Erbα* gene expression by 7.5% lowers the circadian period from 24 h to 23.4 h, in line, qualitatively, with the change observed in the experiments. However, in the model, for parameter values considered in this work, a complete removal of REV-ERBα abolishes the oscillations. This result seems to contradict the findings of Preitner *et al.* [82], but fits with the observation that depletion of both REV-ERBα and REV-ERBβ, a closely related form of the protein, renders mouse embryonic fibroblasts arrhythmic [83]. In the model, we do not distinguish between REV-ERBα and REV-ERBβ and consider a single form of the protein.

A spectrum of possible consequences of bidirectional interactions, depending on the relative strengths of coupling, may explain the apparently conflicting observations that the circadian period is locked to that of the cell cycle in proliferating NIH 3T3 fibroblast cells in cultures [37,38], but progressively changes as cells reach confluence and the cell cycle stops, while the cell cycle and the circadian clock are disconnected in some other fibroblast cell lines [19]. The multiplicity of possible outcomes of bidirectional coupling pertains to abnormal cell proliferation in cancer, given that a loss of connection between the circadian clock and the cell cycle promotes tumour growth [34,84–87], while, in reverse, inhibition of tumour growth is observed [88] upon enhancement of circadian clock function in cancer cells.

The importance of the synchronization between the cell cycle and the circadian clock was already established in unicellular organisms such as cyanobacteria, in which DNA replication occurs optimally at a certain phase of the LD cycle so as to avoid the mutagenic effect of free radicals [2–4]. In multicellular organisms, the coupling of the cell cycle and the circadian clock probably allows synchronization of the former with the circadian variation of metabolic processes [8–10]. Although its molecular bases remain to be established, the protective role of the circadian clock against tumour proliferation reflects the physiological importance of synchronization of the cell cycle and the circadian clock at the cellular and tissue levels. Another indication of the role of the coupling between cell proliferation and the circadian clock *in vivo* is provided by the observation that liver generation is impaired in arrhythmic *Cry*-deficient mice [23]. The circadian clock is known to control the majority of cellular processes [14,15]. Even if the coupling mechanisms between the circadian clock and the cell cycle might have other functional roles than providing links for their synchronization, the present study shows that bidirectional coupling of the two rhythms readily leads to their robust synchronization and avoids the occurrence of complex, irregular oscillations in the cell cycle. Such irregular oscillations, which often occur outside the domain of entrainment in the case of unidirectional coupling [56], might have detrimental consequences for the cell because they would likely perturb the normal operation of the cell cycle. This prediction, of physiological import, represents a major result of the present study, which examined in a comprehensive manner the dynamical consequences of bidirectional coupling of the two cellular rhythms.

## 4. Models

Links to the evolution equations, definition of parameters and parameter values for the models for the circadian clock and the cell cycle in mammalian cells are given in the electronic supplementary material, together with the additional equations describing the different modes of coupling of the circadian clock and the cell cycle, the initial conditions and the computer code used in numerical simulations.

## Supplementary Material

Supporting Information

## Data accessibility

Additional data are given in the electronic supplementary material.
