# Supplementary material for: Robust synchronization of the cell cycle and the circadian clock through bidirectional coupling
Source: J R Soc Interface. 2019 Sep 11;16(158):20190376. doi: 10.1098/rsif.2019.0376 (PMC6769306; doi:10.1098/rsif.2019.0376)
Supplement: Supporting Information [file rsif20190376supp1.docx]

**Supporting information**

**Robust synchronization of cell cycle and circadian clock**

**through bidirectional coupling**

Jie Yan and Albert Goldbeter

**1. Model for bidirectional coupling of cell cycle and circadian clock** …..………………… p.1

**2. Coupling the cell cycle to the circadian clock via induction of *Wee1* by BMAL1** ……... p.2

**3. Coupling the circadian clock to the cell cycle via phosphorylation of REV-ERBα
by Cyclin B/CDK1** …………..………………………………………………………………... p.3

**4. Coupling the cell cycle to the circadian clock via indirect repression of *Cyclin E*
by BMAL1** ………………………………………………………………….…………….…… p.3

**5. Coupling the circadian clock to the cell cycle via inhibition by
Cyclin B/CDK1 of transcription at mitosis** ……………………………………………….. p.4

**6. Coupling the circadian clock to light-dark (LD) cycle by inducing the
transcription of *Per* mRNA** …………………………………..……………………………..... p.4

**7. Coupling the circadian clock to the cell cycle via putative phosphorylation of**
**BMAL1 by Cyclin B/CDK1** ……………………………………………………………...…… p.5

**8.** **Variables and parameters in Eqs. (1) ­­­– (11)** ……………………………………………… p.5

**9. Initial conditions for numerical simulations** ………………...…………………………. p.11

**10. Figures S1-S9** ………………………………………………...……………………………. p.13

**11.** **Computer code for numerical simulations of the model for bidirectional coupling
of cell cycle and circadian clock** ………...………………………………………………..… p.24

**12. References for Supporting Informations** ………...…………………………………….... p.29

**1. Model for bidirectional coupling of cell cycle and circadian clock**

Using detailed computational models previously proposed for the mammalian circadian clock (SI Ref 1) and the mammalian cell cycle (SI Ref 2), and following the approach developed in (SI Ref 3), we considered different implementations for the coupling of these two biological oscillators, as detailed below in sections 2-7.

The evolution equations of the model for the mammalian circadian clock are listed in the Supporting Information of (SI Ref 1); see:

www.pnas.org/content/100/12/7051/tab-figures-data.

The definitions and values for the parameters of the circadian clock model are listed in the legend to Fig. 8 at the same address, with *K*_ib_ =1 nM, *k*_9_=0.63h^-1^.

The evolution equations of the model for the CDK network driving the mammalian cell cycle are listed as eqs. [1]-[39] in Supporting Information of (SI Ref 2); see: www.pnas.org/content/106/51/21643/tab-figures-data.

The definition and values of the parameters for this model are listed in Table S2 at the same address, with *v_cb_*=0.055μM h^-1^.

The autonomous period of oscillations in the circadian clock and cell cycle models can be altered through the scaling parameters *delta* and *eps* which multiply the evolution equations in these two models, respectively.

**2. Coupling the cell cycle to the circadian clock via induction of *Wee1* by BMAL1**

A large number of experiments have shown that the mammalian circadian clock can regulate the levels of some proteins involved in the control of the cell cycle, such as WEE1, Cyclin E, Cyclin B, and p21. In this manuscript, following our previous approach (SI Ref 3), we mainly considered the coupling of the cell cycle to the circadian clock via WEE1, a protein kinase that inhibits Cyclin-dependent kinases such as CDK1 (SI Ref 4).

Experimental data showed that the CLOCK/BMAL1 complexes can induce the transcription of *Wee1* mRNA via binding to E-box (SI Ref 5). As in (SI Ref 3), we incorporated *Wee1* mRNA as a new variable into the model of cell cycle to describe its coupling to the circadian clock. The time evolutions of the concentrations of *Wee1* mRNA (*M*w) and WEE1 protein are governed by eqs. (1) and (2) below. The rate of synthesis of *Wee1* mRNA consists of two terms. The first term, *v_swee1_*, represents the basal rate of synthesis of *Wee1* mRNA. The second term, *v*_sw_, denotes the rate of synthesis of *Wee1* mRNA that depends on nuclear CLOCK/BMAL1 (*B*n). Parameter *v*_sw_ measures the strength of coupling of the cell cycle to the circadian clock.

$\frac{dMw}{dt}=v_{swee1}+v_{sw}\frac{{B_{n}}^{nmw}}{{K_{aw}}^{nmw}+{B_{n}}^{nmw}}-v_{dmw}\frac{Mw}{K_{dmw}+Mw}$ (1)

$\frac{dWee1}{dt}={(k}_{sw}Mw-V_{m7b}\left( Mb+i_{b} \right)\frac{Wee1}{K_{7b}+Wee1}+V_{m8b}\frac{{Wee1}_{p}}{K_{8b}+{Wee1}_{p}}-k_{dwee1}Wee1)eps$ (2)

The coupling via *Wee1* in eqs. (1) and (2) was slightly modified with respect to our previous study of coupling the cell cycle to the circadian clock in (SI Ref 3). Here, we assumed that *v_swee1_* is

the basal rate of *Wee1* mRNA synthesis, rather than the basal rate of synthesis of the WEE1 protein. The present description is more faithful because the basal synthesis of WEE1, which is independent of the circadian clock, also depends on *Wee1* mRNA. Parameter values in eqs. (1) and (2), as in subsequent eqs. (3)-(11), are listed in Table S2 in Section 8.

**3. Coupling the circadian clock to the cell cycle via phosphorylation of REV-ERBα by Cyclin B/CDK1**

Recent experimental evidence indicates that the cell cycle regulates the circadian clock. Thus, CDK1 phosphorylates the protein REV-ERBα which mediates negative autoregulation of *Bmal1* expression in the circadian clock; phosphorylated REV-ERBα is recognized by protein FBXW7, which targets it to the proteasome (SI Ref. 6). The Cyclin B/CDK1 complex thus reduces REV-ERBα levels through phosphorylation, which marks the protein for degradation. To incorporate this link into the model we added two kinetic equations for phosphorylated REV-ERBα in cytoplasm and nucleus. The time evolution of the concentrations of REV-ERBα proteins are governed by eqs. (3)-(6). Parameter *V*_Cdk1_ measures the strength of coupling of the circadian clock to the cell cycle:

$\frac{dRc}{dt}=k_{\mathrm{sR}}Mr-k_{9}Rc+k_{10}Rn-{(V}_{1R}+V_{Cdk1}Mb)\frac{Rc}{K_{p}+Rc}+V_{2R}\frac{R\mathrm{cp}}{K_{\mathrm{dp}}+R\mathrm{cp}}-k_{dn}Rc$ (3)

$\frac{dRcp}{dt}={(V}_{1R}+V_{Cdk1}Mb)\frac{Rc}{K_{p}+Rc}-V_{2R}\frac{R\mathrm{cp}}{K_{\mathrm{dp}}+R\mathrm{cp}}-v_{\mathrm{dRC}}\frac{R\mathrm{cp}}{K_{d}+R\mathrm{cp}}-k_{dn}R\mathrm{cp}$ (4)

$\frac{dRn}{dt}=k_{9}Rc-k_{10}Rn-{(V}_{3R}+V_{Cdk1}Mb)\frac{Rn}{K_{p}+Rn}+V_{4R}\frac{R\mathrm{cp}}{K_{\mathrm{dp}}+R\mathrm{cp}}-k_{dn}Rn$ (5)

$\frac{dR\mathrm{np}}{dt}={(V}_{3R}+V_{Cdk1}Mb)\frac{Rn}{K_{p}+Rn}-V_{4R}\frac{R\mathrm{np}}{K_{\mathrm{dp}}+R\mathrm{np}}-v_{\mathrm{dRN}}\frac{R\mathrm{np}}{K_{d}+R\mathrm{np}}-k_{dn}R\mathrm{np}$ (6)

Parameters $V_{1R}$ and $V_{3R}$ denotes the basal rates of phosphorylation of cytoplasmic and nuclear REV-ERBα, while $V_{Cdk1}Mb$ represents the maximum rate of phosphorylation of cytoplasmic and nuclear REV-ERBα by Cyclin B/CDK1.

**4. Coupling the cell cycle to the circadian clock via indirect repression of *Cyclin E* by BMAL1**

We also considered the coupling of the cell cycle to the circadian clock via Cyclin E. Experimental data show that BMAL1 can indirectly repress the expression of *Cyclin E* by promoting its inhibitor c-MYC (SI Refs 7 and 8). To incorporate this coupling into the model, we follow the approach proposed in (SI Ref 3) and add an equation to represent the temporal evolution of *Cyclin E* mRNA. Equations (7) and (8) [which are identical to eqs. (5) and (6) in (SI Ref. 3)] describe the time evolution of the concentrations of *Cyclin E* mRNA (*MCe*) and Cyclin E protein (*Ce*). For simplicity
we consider that BMAL1 directly inhibits the rate of synthesis of *Cyclin E* mRNA, *v_sce_*, which measures the strength of this coupling of the cell cycle to the circadian clock (the coupling strength could also be measured by the inhibition constant *K*_ice_):

$\frac{dMCe}{dt}=v_{sce}\frac{{K_{ice}}^{nce}}{{K_{ice}}^{nce}+{B_{n}}^{nce}}-V_{dmce}\frac{Mce}{K_{dmce}+Mce}$ (7)

$\frac{dCe}{dt}={(k}_{ce}E2F\frac{K_{i9}}{K_{i9}+pRB}\frac{K_{i10}}{K_{i10}+pRB_{p}}+k_{ce2}Mce-k_{com2}Ce\left( Cdk2_{tot}-\left( Mei+Me+Mep27+Mai+Ma+Map27 \right) \right)+k_{decom2}Mei-V_{de}\frac{Skp2}{K_{dceskp2}+Skp2}\frac{Ce}{K_{de}+Ce}-k_{dde}Ce)\cdot eps$ (8)

**5. Coupling the circadian clock to the cell cycle via inhibition by Cyclin B/CDK1 of transcription at mitosis**

The experimental data of Bieler et al (SI Ref. 9) support the view that the cell cycle can control the circadian clock via inhibition of transcription during mitosis. This inhibition is mediated through phosphorylation of a protein of the transcription machinery by Cyclin B/CDK1 (SI Ref. 10). To describe the inhibition by CDK1 of transcription processes during mitosis we add the term ($v_{\mathrm{in}}\frac{{K_{Icdk1}}^{ncdk1}}{{K_{Icdk1}}^{ncdk1}+{Mb}^{ncdk1}}$) in the equations that govern, in the circadian clock model, the time evolution of *Per*, *Cry1*, *Bmal1* and *Rev-Erbα* mRNAs (see [www.pnas.org/content/100/12/7051/tab-figures-data](http://www.pnas.org/content/100/12/7051/tab-figures-data)). This simplified phenomenological representation of the inhibitory effect of Cyclin B/CDK1 (*Mb*) takes the form of an inhibitory function of the Hill type, characterized by a degree of cooperativity *ncdk1*. The choice of a Hill function allows for cooperativity when *ncdk1*>1. Such cooperativity could correspond to the possible occurrence of zero-order ultrasensitivity (SI Ref 11) in phosphorylation by CDK1.

**6. Coupling the circadian clock to LD cycle by inducing the transcription of *Per* mRNA**

Experiments indicate that light controls the circadian clock by promoting the expression of the Per genes in mammals (SI Ref 12) We incorporate the effect of light by modifying the form of the maximum rate of Per expression, v_sP_. Parameter L represents the incremental rate of *Per* mRNA expression due to the effect of light. As in (SI Ref 1) it varies as a square wave, going from 0 during the dark phase to a higher constant value, v*_sPmax_*, during the light phase.

$\frac{dMp}{dt}=(L+v_{sP})\frac{{Bn}^{n}}{{K_{\mathrm{AP}}}^{n}+{Bn}^{n}}-v_{\mathrm{mP}}\frac{Mp}{K_{\mathrm{mP}}+Mp}-k_{dmp}Mp$ (9)

**7. Coupling the circadian clock to the cell cycle via putative phosphorylation of BMAL1 by Cyclin B/CDK1**

To investigate another mode of coupling of the circadian clock to the cell cycle, we consider the possibility that BMAL1 is phosphorylated by CDK1. Such regulation has not been observed experimentally, but CDK5, another member of the CDK family, active in neuronal processes, was found to phosphorylate CLOCK (SI Ref 13). The putative phosphorylation of BMAL1 (which makes a complex with CLOCK) introduces a negative feedback loop, because CDK1 would lower the level of BMAL1 by marking it for degradation. As we consider that BMAL1 induces *Wee1* and thereby increases the level of WEE1, which is an inhibitor of CDK1, the situation corresponds to mutual inhibition of the cell cycle and the circadian clock, which situation leads to antiphase oscillations.

To describe this coupling of the circadian clock to the cell cycle, we assume that Cyclin B/CDK1 can phosphorylate the cytoplasmic BMAL1 protein. This coupling is incorporated in the model by an addition phosphorylation for the cytoplasmic BMAL1 protein, at a rate proportional to the concentration of Cyclin B/ CDK1 (*Mb*). Equations (10) and (11) describe the coupling of the circadian clock to the cell cycle. The value of *V^’^_Cdk1_* measures the coupling strength of the circadian clock to the cell cycle.

$\frac{dBc}{dt}=k_{sB}Mbmal1-\left( V_{1B}+V_{Cdk1}^{’}Mb \right)\frac{Bc}{K_{p}+Bc}+V_{2B}\frac{{Bc}_{p}}{K_{dp}+{Bc}_{p}}-k_{5}Bc+k_{6}Bn-k_{dn}Bc$ (10)

$\frac{dBcp}{dt}={(V}_{1B}+V_{Cdk1}^{’}Mb)\frac{Bc}{K_{p}+Bc}-V_{2B}\frac{{Bc}_{p}}{K_{dp}+{Bc}_{p}}-v_{dBc}\frac{{Bc}_{p}}{K_{d}+{Bc}_{p}}-k_{dn}{Bc}_{p}$ (11)

**8.** **Variables and parameters in Eqs. (1) ­­­– (11)**

Variables in Eqs. (1) – (11) are defined in Table S1 below, while the definitions and numerical values of the parameters which appear in these equations are listed in Table S2 below.

Other variables and parameters that appear in the remaining equations of the models for the circadian clock and cell cycle are listed, together with these equations, at the links given in Section 1 of the present Supporting Informations.

**Table S1: Definitions of variables in Equations (1)-(11).**

| **Concentration variables which appear in Eq. (1) – (11)** | |
| --- | --- |
| **Symbol** | **Definition** |
| $Mw$ | *Wee1* mRNA |
| $B_{n}$ | Nuclear CLOCK/BMAL1 complex |
| $Wee1$ | Unphosphorylated WEE1 |
| $Mb$ | Active Cylin B/CDK1 |
| ${Wee1}_{p}$ | Phosphorylated WEE1 |
| $Mr$ | *Rev-Erbα* mRNA |
| $Rc$ | Unphosphorylated REV-ERBα in cytoplasm |
| $Rcp$ | Phosphorylated REV-ERBα in cytoplasm |
| $Rn$ | Unphosphorylated REV-ERBα in nucleus |
| $R\mathrm{np}$ | Phosphorylated REV-ERBα in nucleus |
| $MCe$ | *Cyclin E* mRNA |
| $Ce$ | Free Cyclin E |
| $E2F$ | Transcription factor E2F |
| $pRB$ | Unphosphorylated Retinoblastoma protein |
| $pRB_{p}$ | Phosphorylated Retinoblastoma protein |
| $Mei$ | Inactive complex Cyclin E/CDK2 |
| $Me$ | Active complex Cyclin E/CDK2 |
| $Mep27$ | Complex Cyclin E/CDK2/p27 |
| $Mai$ | Inactive complex Cyclin A/CDK2 |
| $Ma$ | Active complex Cyclin A/CDK2 |
| $Map27$ | Complex Cyclin A/CDK2/p27 |
| $Skp2$ | F-box protein Skp2 |
| $Mp$ | *Per* mRNA |
| $Mbmal1$ | *Bmal1* mRNA |
| $Bc$ | Unphosphorylated cytoplamic BMAL1 |
| ${Bc}_{p}$ | Phosphorylated cytoplamic BMAL1 |

**Table S2: Definitions and numerical values of parameters in Equations (1)-(11).**

| **Coupling the cell cycle to the circadian clock via induction of *Wee1* by BMAL1:**  **Eqs. (1) ­– (2)** | | |
| --- | --- | --- |
| **Symbol** | **Definition** | **Value** |
| *v_sw_* | Rate of synthesis of *Wee1* mRNA that depends on nuclear CLOCK/BMAL1 (*B*n) | Control parameter: changes with coupling strength |
| *v_swee1_* | Basal synthesis rate of *Wee1* mRNA | 0.0117 μM·h^-1^ |
| *k_sw_* | Rate of WEE1 synthesis | 5 h^-1^ |
| *V_m7b_* | Rate constant for inactivation of kinase WEE1 through phosphorylation by Cylin B/CDK1 and other kinases | 1.2 h^-1^ |
| *V_m8b_* | The maximum rate of kinase WEE1 activation through dephosphorylation | 1 μM·h^-1^ |
| *i_b_* | Factor measuring the contribution of kinases other than Cdk1 to phosphorylation and inactivation of kinase WEE1 | 0.75 μM |
| *K_7b_* | Michaelis constant for Wee1 inactivation through phosphorylation by Ccylin B/CDK1 and other kinases | 0.1 μM |
| *K_8b_* | Michaelis constant for WEE1 activation through dephosphorylation | 0.1 μM |
| *K_aw_* | Michaelis constant for activation of *Wee1* mRNA by CLOCK/BMAL1 (Bn) | 2 nM |
| *K_dmw_* | Michaelis constant for *Wee1* mRNA degradation | 0.5 μM |
| *nmw* | Degree of cooperativity for activation by CLOCK/BMAL1 (Bn) of *Wee1* mRNA (*Mw*) synthesis | 4 |
| *V_dmw_* | Maximum degradation rate of *Wee1* mRNA | 0.5 μM·h^-1^ |
| *k_dwee1_* | Degradation rate of WEE1 | 0.1 μM·h^-1^ |
| **Coupling the circadian clock to the cell cycle via phosphorylation of REV-ERBα  by Cyclin B/CDK1: Eqs. (3) – (6)** | | |
| **Symbol** | **Definition** | **Value** |
| *V*_Cdk1_ | Rate of phosphorylation of REV-ERBα dependent on CYCLIN B/CDK1 | Control parameter: changes with coupling strength |
| *k_sR_* | Rate of synthesis of cytoplasmic REV-ERBα | 1.7 nM·h^–1^ |
| *k_9_* | Rate constant for transfer of REV-ERBα from cytoplasm to nucleus | 0.63 h^–1^ |
| *k_10_* | Rate constant for transfer of REV-ERBα from nucleus to cytoplasm | 0.4 h^–1^ |
| *V_1R_* | Rate of phosphorylation of cytoplasmic REV-ERBα independent of Cyclin B/CDK1 | 4 nM·h^–1^ |
| *V_2R_* | Rate of dephosphorylation of cytoplasmic REV-ERBα | 8 nM·h^–1^ |
| *V_3R_* | Rate of phosphorylation of nuclear REV-ERBα independent of Cyclin B/CDK1 | 8 nM·h^–1^ |
| *V_4R_* | Rate of dephosphorylation of cytoplasmic REV-ERBα | 4 nM·h^–1^ |
| *K*_p_ | Michaelis constant for phosphorylation of REV-ERBα | 1.006 nM |
| *K*_dp_ | Michaelis constant for dephosphorylation of REV-ERBα | 0.1 nM |
| *K*_d_ | Michaelis constant for degradation of REV-ERBα | 0.3 nM |
| *v*_dRC_ | Maximum rate of degradation of cytoplasmic phosphorylated REV-ERBα | 4.4 nM·h^–1^ |
| *v*_dRN_ | Maximum rate of degradation of nuclear phosphorylated REV-ERBα | 0.8 nM·h^–1^ |
| *k_dn_* | Apparent first-order rate constant for non-specific REV-ERBα degradation | 0.02 h^–1^ |
| **Coupling the cell cycle to the circadian clock via indirect repression of *Cyclin E*  by BMAL1: Eqs. (7) – (8)** | | |
| **Symbol** | **Definition** | **Value** |
| *v_sce_* | Maximum rate of synthesis of *Cyclin E* mRNA dependent on the indirect repression of CLOCK/BMAL1 (*Bn*) | Control parameter: changes with coupling strength |
| *k_ce_* | Rate of synthesis of Cyclin E induced by E2F | 0.29 h^-1^ |
| *k_ce2_* | Rate of synthesis of Cyclin E mediated indirectly by CLOCK/BMAL1 | 5 h^-1^ |
| *k_com2_* | Association rate of complex between Cyclin E and CDK2 | 0.2 μM^-1^·h^-1^ |
| *k_decom2_* | Dissociation rate of complex between Cyclin E and CDK2 | 0.1 h^-1^ |
| *Cdk2_tot_* | Total concentration of CDK2 | 2 μM |
| *K_ice_* | Inhibition constant for repression of *Cyclin E* mRNA by CLOCK/BMAL1(*Bn*) | 1 μM |
| *K_dmce_* | Michaelis constant for degradation of *Cyclin E* mRNA | 0.5 μM |
| *K_i9_* | Michaelis constant for inhibition by pRB of Cyclin E synthesis | 0.1 μM |
| *K_i10_* | Michaelis constant for inhibition by phosphorylated pRB of Cyclin E synthesis | 2 μM |
| *K_dceskp2_* | Michaelis constant for activation by Skp2 of Cyclin E degradation | 2 μM |
| *K_de_* | Michaelis constant for Cyclin E degradation | 0.1 μM |
| *nce* | Degree of cooperativity for repression by CLOCK/BMAL1 (*Bn*) of *Cyclin E* mRNA (*Mce*) synthesis | 4 |
| *V_dmce_* | Maximum rate of degradation of *Cyclin E* mRNA | 0.5 μM·h^-1^ |
| *V_de_* | Maximum rate of degradation of Cyclin E elicited by Skp2 | 3 μM·h^-1^ |
| *k_dde_* | Apparent first-order rate constant for non-specific Cyclin E degradation | 0.005 h^-1^ |
| **Coupling the circadian clock to the cell cycle via inhibition by Cyclin B/CDK1  of transcription at mitosis (Supporting Informations, Section 5)** | | |
| **Symbol** | **Definition** | **Value** |
| *v_in_* | Maximum rate of synthesis of mRNAs inhibited by Cyclin B/CDK1 at mitosis | 0.7 nM·h^–1^ |
| *K_Icdk1_* | Inhibition constant for repression by Cyclin B/CDK1 at mitosis | 0.5 nM |
| *ncdk1* | Degree of cooperativity for repression by Cyclin B/CDK1 at mitosis | 2 in Fig.9 and 1 in Fig. S7 |
| **Coupling the circadian clock to LD cycle by inducing the transcription of *Per* mRNA: Eq. (9)** | | |
| **Symbol** | **Definition** | **Value** |
| *L* | Rate of synthesis of *Per* mRNA that depends on light | Square wave |
| *v_sPmax_* | Maximum rate of synthesis of *Per* mRNA that depends on light intensity | Control parameter: changes with coupling strength |
| *v_sP_* | Basal rate of synthesis of *Per* mRNA | 2.4 nM·h^–1^ |
| *v_mP_* | Maximum rate of degradation of *Per* mRNA | 2.2 nM·h^–1^ |
| *K_mP_* | Michaelis constant for *Per* mRNA degradation | 0.3 nM |
| *K_AP_* | Activation constant for induction of *Per* mRNA by CLOCK/ BMAL1 | 0.6 nM |
| *n* | Degree of cooperativity for the activation by CLOCK/BMAL1 of *Per* mRNA induction | 2 |
| *k_dmp_* | Apparent first-order rate constant for non-specific *Per* mRNA degradation | 0.02 h^–1^ |
| **Coupling the circadian clock to the cell cycle via putative phosphorylation of BMAL1 by Cyclin B/CDK1: Eqs. (10) – (11)** | | |
| **Symbol** | **Definition** | **Value** |
| *V^’^_Cdk1_* | Rate of phosphorylation of cytoplasmic BMAL1 by Cyclin B/CDK1 | Control parameter: changes with coupling strength |
| *k_sB_* | The synthesis rate of cytoplasmic BMAL1 | 0.32 h^–1^ |
| *V_1B_* | The basal phosphorylation rate of cytoplasmic BMAL1 | 1.4 nM·h^–1^ |
| *V_2B_* | The dephosphorylation rate of cytoplasmic BMAL1 | 0.2 nM·h^–1^ |
| *v_dBc_* | The maximum degradation rate of phosphorylated BMAL1 | 3 nM·h^–1^ |
| *K_p_* | Michaelis constant for cytoplasmic BMAL1 phosphorylation | 1.006 nM |
| *K_dp_* | Michaelis constant for cytoplasmic BMAL1 dephosphorylation | 0.1 nM |
| *K_d_* | Michaelis constant for degradation of phosphorylatyed BMAL1 | 0.3 nM |
| *k_5_* | The transfer rate of BMAL1 from cytoplasm to nucleus | 0.8 h^–1^ |
| *k_6_* | The transfer rate of BMAL1 from nucleus to cytoplasm | 0.4 h^–1^ |
| *k_dn_* | Apparent first-order rate constant for non-specific BMAL1 degradation | 0.02 nM·h^–1^ |

* * * * *

**9. Initial condition for numerical simulations**

The differential equations that govern the time evolution of the model were integrated numerically by means of the Runge-Kutta method provided in the program XPPAUTO developed by Dr. Bard Ermentrout (SI Ref 14). Initial conditions for the figures in the main text and in Supporting information are listed below (as in the original publications, concentration units are expressed tentatively in nM for circadian variables [see Supporting information in (SI Ref 3)] and in μM for cell cycle variables [see Supporting information in (SI Ref 3)]).

Initial conditions for Figs. 2-8, 9c and 10, as well as Figs. S2-S6, S7d, S8, S9:

Mp=0.1, Mc=0.1, Mbmal=0.1, Pc=0.1, Cc=0.1, Pcp=0.1, Ccp=0.1, PCc=0.1, PCn=0.1, PCcp=0.1, PCnp=0.1, Bc=0.1, Bcp=0.1, Bn=0.1, Bnp=0.1, In=0.1, Mr=0.1, Rc=0.1, Rn=0.1, AP1=0.01, pRB=1, pRBc1=0.25, pRBp=0.1, pRBc2=0.01, pRBpp=0.01, E2F=0.1, E2Fp=0.05, Cd=0.01, Mdi=0.01, Md=0.01, Mdp27=0.01, Ce=0.01, Mei=0.01, Me=0.01, Skp2=0.01, Mep27=0.01, Pei=0.01, Pe=0.01, Ca=0.01, Mai=0.01, Ma=0.01, Map27=0.01, p27=0.25, p27p=0.01, Cdh1i=0.01, Cdh1a=0.01, Pai=0.01, Pa=0.01, Cb=0.01, Mbi=0.01, Mb=0.01, Mbp27=0.01, Cdc20i=0.01, Cdc20a=0.01, Pbi=0.01, Pb=0.01, Mw=0, Wee1=0.1, Wee1p=0.01

Initial conditions for Fig. 9d:

Mp=0.10066, Mc=0.14857, Mbmal=1.115, Pc=0.002765, Cc=54.7852, Pcp=0.0015232, Ccp=0.72264, PCc=0.044913, PCn=0.01699, PCcp=0.01678, PCnp=0.0062605, Bc=0.21504, Bcp=0.022324, Bn=0.1302, Bnp=0.01218, In=0.00040994, Mr=0.19024, Rc=1.1577, Rcp=0.028306, Rn=1.2007, Rnp=17.4551, AP1=6.0606, pRB=1.4226, pRBc1=0.57566, pRBp=12.4142, pRBc2=2.5086, pRBpp=0.00083091, E2F=4.1817, E2Fp=0.013621, Cd=0.094089, Mdi=0.021248, Md=0.70314, Mdp27=0.64075, Mce=0, Ce=0.00044067, Mei=0.0047019, Me=0.01663, Skp2=12.4626, Mep27=0.012486, Pei=0.17561, Pe=1.3909, Ca=0.0027318, Mai=0.025911, Ma=0.0090928, Map27=0.0054701, p27=0.41494, p27p=0.017831, Cdh1i=0.54582, Cdh1a=0.0079194, Pai=0.59617, Pa=0.23363, Cb=0.78924, Mbi=0.033587, Mb=0.36915, Mbp27=0.080951, Cdc20i=0.034632, Cdc20a=1.8889, Pbi=0.073905, Pb=1.0562, Mw=0.012069, Wee1=0.12688, Wee1p=0.23768

Initial conditions for Fig. 11:

Mp=0.1,Mc=0.1, Mbmal=0.1, Pc=0.1,Cc=0.1,Pcp=0.1, Ccp=0.1, PCc=0.1, PCn=0.1, PCcp=0.1, PCnp=0.1, Bc=0.1, Bcp=0.1, Bn=0.1, Bnp=0.1, In=0.1, Mr=0.1, Rc=0.1, Rn=0.1, AP1=0.01, pRB=1, pRBc1=0.25, pRBp=0.1, pRBc2=0.01, pRBpp=0.01, E2F=0.1, E2Fp=0.05, Cd=0.01, Mdi=0.01, Md=0.01, Mdp27=0.01, Ce=0.01, Mei=0.01, Me=0.01, Skp2=0.01, Mep27=0.01, Pei=0.01, Pe=0.01, Ca=0.01, Mai=0.01, Ma=0.01, Map27=0.01, p27=0.25, p27p=0.01, Cdh1i=0.01, Cdh1a=0.01, Pai=0.01, Pa=0.01, Cb=0.01, Mbi=0.01, Mb=0.01, Mbp27=0.01, Cdc20i=0.01, Cdc20a=0.01, Pbi=0.01, Pb=0.01, Mw=0, Wee1=0.1, Wee1p=0.01

Initial conditions for Fig. S7b:

Mp=0.98256, Mc=1.5334, Mbmal=2.417, Pc=0.028353, Cc=54.3483, Pcp=0.01679, Ccp=0.71589, PCc=0.56291, PCn=0.19645, PCcp=0.33109, PCnp=0.083493, Bc=0.66874, Bcp=0.058477, Bn=0.44064, Bnp=0.036191, In=0.01706, Mr=1.7536, Rc=5.0957, Rcp=0.050954, Rn=2.7461, Rnp=10.054, AP1=6.0606, pRB=1.4642, pRBc1=1.1295, pRBp=12.4611, pRBc2=4.8084,

pRBpp=0.0051185, E2F=7.8025, E2Fp=0.055843, Cd=0.094109, Mdi=0.022562, Md=1.1583, Mdp27=0.18297, Mce=0Ce=0.013178, Mei=0.030955, Me=0.098431, Skp2=0.11394, Mep27=0.0089476, Pei=0.098981, Pe=1.529, Ca=0.082436, Mai=0.2562, Ma=0.028919, Map27=0.0015649, p27=0.045078, p27p=0.043375, Cdh1i=0.0041081, Cdh1a=1.0922, Pai=0.54609, Pa=0.30008, Cb=0.53144, Mbi=0.28073, Mb=0.017077, Mbp27=0.00047442, Cdc20i=0.70637, Cdc20a=0.02038, Pbi=0.54419, Pb=0.11075, Mw=0.020105, Wee1=0.53936, Wee1p=0.26349.

Initial conditions for Fig. S7c:

Mp=0.076123, Mc=0.11289, Mbmal=0.94931, Pc=0.0024184, Cc=46.5114, Pcp=0.0013377, Ccp=0.71481, PCc=0.034429, PCn=0.013627, PCcp=0.012908, PCnp=0.0050582, Bc=0.17939, Bcp=0.018917, Bn=0.10892, Bnp=0.010304, In=0.00027939, Mr=0.14423, Rc=1.0842, Rcp=0.02725, Rn=1.1764, Rnp=10.8059, AP1=6.0606, pRB=1.5549, pRBc1=0.12823, pRBp=12.9731, pRBc2=0.53496, pRBpp=0.008884, E2F=0.84716, E2Fp=0.11619, Cd=0.094085, Mdi=0.022742, Md=1.2588, Mdp27=0.080219, Mce=0Ce=0.00014849, Mei=0.01997, Me=0.16469,

Skp2=15.3111, Mep27=0.0083879, Pei=0.07249, Pe=1.5948, Ca=0.0038995, Mai=0.052378, Ma=0.24918,Map27=0.0078512,p27=0.025584, p27p=0.019133, Cdh1i=0.54837, Cdh1a=0.0031393, Pai=0.12689,Pa=1.1688,Cb=1.3401,Mbi=0.033052,Mb=0.45069,Mbp27=0.0065493,Cdc20i=0.026772, Cdc20a=1.9105, Pbi=0.057553, Pb=1.085, Mw=0.012055, Wee1=0.11165, Wee1p=0.24596.

**10.** **Figures S1-S9 for Supporting Informations**

Fig. S1: Unidirectional versus bidirectional coupling of cell cycle and circadian clock. The highly simplified schemes show: (a) unidirectional coupling of cell cycle to circadian clock via the induction by BMAL1 of the gene *Wee1* coding for the kinase WEE1, which inhibits Cyclin B/CDK1 through phosphorylation; (b) unidirectional coupling of circadian clock to cell cycle via the phosphorylation by Cyclin B/CDK1 of REV-ERBα, which leads to the enhanced degradation of this protein, which represses the expression of *Bmal1*; (c) bidirectional coupling of cell cycle and circadian clock, incorporating the two modes of coupling shown in (a) and (b). Indicated are the parameters *v*_sw_ and *V*_Cdk1_, which mediate the coupling of the cell cycle to the circadian clock and of the circadian clock to the cell cycle, respectively. Additional modes of coupling (not shown) are considered in the text. More detailed representations of the circadian clock and cell cycle models are shown in Fig. 1.

14.

Fig. S2: Failure of synchronization of cell cycle and circadian clock in the form of simple periodic oscillations in conditions of bidirectional coupling. Shown on the diagram reproduced from Fig. 5a are the points marked *a*, *b*, *c*, *d, e, f, g, h* for which time series for Cyclin B/CDK1 and nuclear REV-ERBα are shown in panels (a)-(h) in Fig. S3. The cell cycle is coupled to the circadian clock via BMAL1-induction of *Wee1* while the circadian clock is linked to the cell cycle through REV-ERBα phosphorylation by CDK1.

Fig. S3: Failure of synchronization of cell cycle and circadian clock, and synchronization in the form of period-2 or period-3 oscillations, in conditions of bidirectional coupling. Shown are the time series for Cyclin B/CDK1 (in red) and nuclear REV-ERBα (in blue). Panels (a)-(h) correspond to points marked *a*, *b*, *c*, *d, e, f, g, h* in the diagram of Fig. S2. (a) At low values of the two coupling strengths, the cell cycle and the circadian clock oscillate independently at their autonomous period of 20h and 24h, respectively. (b) Once the coupling strengths begin to increase, the cell cycle and the circadian clock still fail to synchronize but their periods are slightly modulated. Thus the cell cycle period varies from 19.8h to 20.4h, while the circadian period varies from 23.7h to 24.5h. (c) In the white hole located near the middle of the diagram in Fig. 5a, i.e. at larger values of the coupling coefficients, the circadian clock and the cell cycle both oscillate with a common period of 40.5h. This synchronized behavior represents period-2 oscillations, with two peaks of distinct amplitude per period for nuclear REV-ERBα at intervals of 20.7h and 19.8h, successively. A similar form of synchronized behavior is observed in (d), in another region of the white region in the core of the diagram Fig. 5a. The circadian clock and the cell cycle then display period-2 oscillations consisting of 2 peaks of distinct amplitude per period with a global period of 40.9h, made of intervals of 20.7h and 20.3h, successively. (e) Another example of period-2 oscillations. The two distinct peaks over a period are separated by distinct intervals, which differ according to the variable considered, as indicated on the figure. However, the sum of these intervals remains the same for all variables and defines the period of the oscillations, which is 50.44h in the case considered. (f) Period-3 oscillations consisting of 3 peaks of distinct amplitude per period. These peaks are separated by distinct intervals, which differ according to the variable considered; the sum of these intervals remains the same for all variables and defines the period of the oscillations, which is 59.1h in the case considered. (g) A situation where the cell cycle is arrested while the circadian clock continues to oscillate. (h) A situation where the circadian clock stops while the cell cycle oscillates. The values of the two coupling strengths *v*_sw_ (in μMh^-1^) and *V*_Cdk1_ (in nMh^-1^) are, respectively, 0.001 and 0.1 in (a), 0.003 and 1 in (b), 0.079 and 125.9 in (c), 0.1 and 63.1 in (d), 3.98 and 100 in (e), 0.005 and 316.2 in (f), 10 and 10 in (g), and 0.001 and 10000 in (h).

Fig. S4: Synchronization of bidirectionally coupled cell cycle and circadian clock when the cell cycle is connected with the circadian rhythm through negative regulation of *Cyclin E* expression by BMAL1, alone (a)-(b) or in conjunction with induction of *Wee1* by BMAL1 (c)-(d). In both cases the circadian clock is linked to the cell cycle through REV-ERBα phosphorylation by CDK1. The values of the coupling strengths *v*_sw_ (in μMh^-1^), *v*_sce_ (in μMh^-1^), and *V*_Cdk1_ (in nMh^-1^) are indicated in the panels. The synchronization period *T*_syn_ is determined as a function of the cell cycle autonomous period *T*_CC_, for an autonomous circadian clock period *T*_CR_ equal to 24h. The horizontal and diagonal dashed lines indicate the loci where *T*_syn_ = *T*_CR_ and *T*_syn_ = *T*_CC_.

Fig. S5: Additional coupling through Cyclin E can transform synchronization in the form of period-2 or period-3 oscillations in the conditions of Figs. 5 and S2 into synchronization in the form of simple periodic oscillations. The cell cycle is coupled to the circadian clock via BMAL1-induction of *Wee1* and, additionally, via *Cyclin E* repression by BMAL11, while the circadian clock is linked to the cell cycle through REV-ERBα phosphorylation by CDK1. (a) When *V*_Cdk1_=125.89 nMh^-1^, *v*_sw_ =0.079 μMh^-1^, period-2 oscillations transform into simple oscillations with a period of 24.17h upon incorporating the additional coupling via Cyclin E. (b) When *V*_Cdk1_=251.19 nMh^-1^, *v*_sw_ =0.005 μMh^-1^, period-3 oscillations can also transform into simple oscillations with a period of 28.78h upon incorporating this additional coupling. The coupling strength via Cyclin E in (a) and (b) is *v*_sce_ =0.01 μMh^-1^.


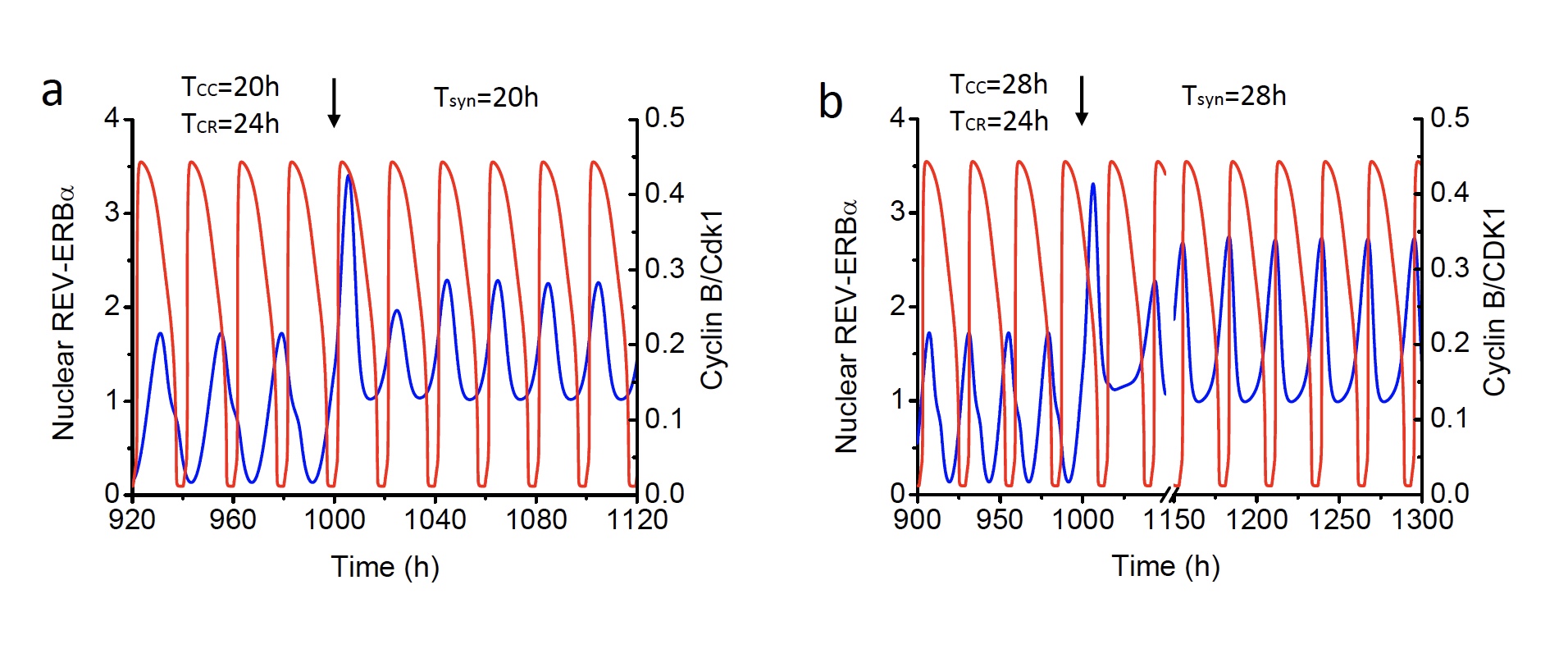


Fig. S6 : Unidirectional coupling of cell cycle to circadian clock via mitotic inhibition of transcription, controlled by CDK1. The time series show entrainment of the circadian clock by the cell cycle when the autonomous period of the cell cycle (*T*_CC_) is 20h (a) or 28h (b), while the autonomous period of the circadian clock (*T*_CR_) is 24h. Unidirectional coupling starts at the time marked by the vertical arrow; before coupling, the cell cycle and circadian clock oscillate independently at their autonomous period. The strength of cell cycle coupling to the circadian clock is measured by parameter *K*_Icdk1_, which is equal to 0.5 μM.

Fig. S7 : Bidirectional coupling and trirhythmicity. Bidirectional coupling is effected via mitotic repression of transcription, controlled by CDK1, and BMAL1-induction of *Wee1*. When the degree of cooperativity *ncdk1* of the inhibition by CDK1 of mRNA synthesis is equal to 1, both birhythmicity and trirhythmicity may occur, depending on the cell cycle autonomous period, *T*_CC_ (a). When *T*_CC_, is 20.5h, the cell cycle and circadian clock (with autonomous period *T*_CR_=24h) may synchronize at a period of 23.74h (b), 30.89h (c) or 25.2h (d), depending on initial conditions (see Section 9 of these Supporting Informations). For smaller or larger values of *T*_CC_, the diagram in (a) predicts the coexistence of two distinct modes of synchronization (birhythmicity), as in Fig. 8 (b)-(d), or synchronization corresponding to a single mode of common oscillations of cell cycle and circadian clock (monorhythmicity).

Fig. S8: Effect of bidirectional coupling when the cell cycle is arrested. (a) Cell cycle arrests when the growth factor (*GF*) decreases from 1 to 0.1. Both the expressions of Cyclin B/CDK1 (red) and Cyclin E/CDK2 (green) are low. Upon bidirectional coupling, when *V*_Cdk1_=100 nM h^-1^ and *v*_sw_=15.8 nM h^-1^, the cell cycle remains arrested. The circadian clock exhibits robust oscillations, with a slightly changed period of 24.4h compared with the autonomous period of T_CR_=24h before coupling. The blue line represents the time evolution of nuclear REV-ERBα. (b) Cell cycle stops when parameter *V*_m1b_, which measures the activation of Cyclin B/CDK1 by phosphatase CDC25, increases from 3.9μM h^-1^ to 10μM h^-1^. Evolution equations in which parameters *GF* and *V*_m1b_ appear in the model for the mammalian cell cycle are listed in the Supporting Informations of SI Ref. 2, at www.pnas.org/content/106/51/21643/tab-figures-data. Cyclin B/CDK1 remains at a high level due to the increased activity of CDC25. Upon bidirectional coupling, when *V*_Cdk1_ = 1 nM h^-1^ and *v*_sw_ = 0.1μM h^-1^, the cell cycle remains arrested while the circadian clock (autonomous period *T*_CR_=24h before coupling) exhibits robust oscillations, with a slightly changed period of 24.24h.


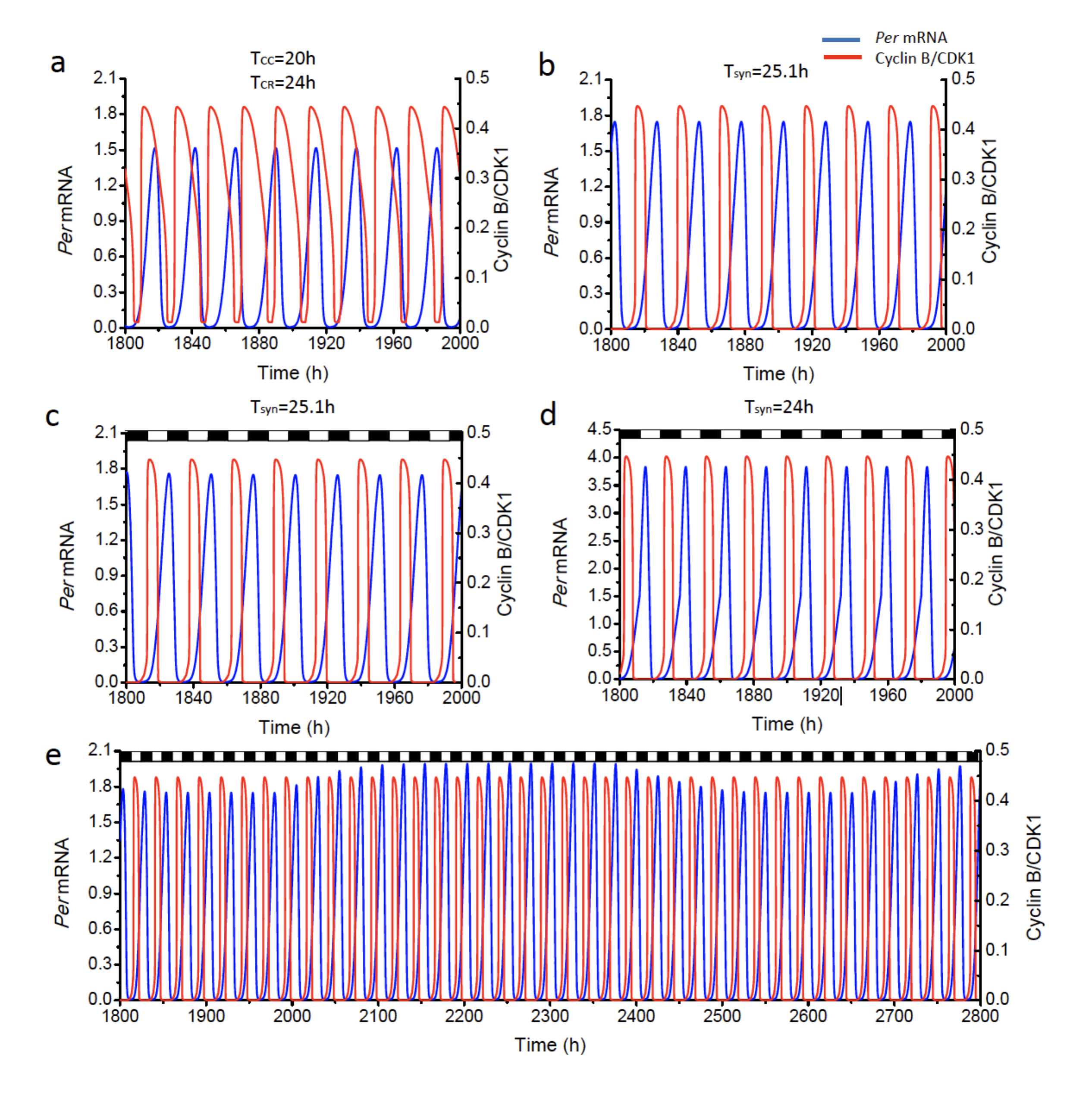


Fig. S9：Bidirectional coupling of cell cycle and circadian clock when the latter is driven by the external light-dark (LD) cycle. (a) Time evolution of *Per* mRNA (blue) and Cyclin B/CDK1 (red) before bidirectional coupling, in the absence of LD cycle. The circadian clock and cell cycle oscillate independently with a period of 24h and 20h, respectively. (b) Time evolution of *Per* mRNA and Cyclin B/Cdk1 after bidirectional coupling, in the absence of LD cycle. The circadian clock and cell cycle are synchronized at a period of 25.1h. (c) When the circadian clock is weakly coupled to the LD cycle, i.e. when parameter L in eq. (9) in Section 6 of Supporting Informations varies as a square wave between 0 in the 12h-dark phase and v*_sPmax_* =0.01 in the 12h-light phase, the LD cycle has little effect on the circadian clock. Then the circadian clock and the cell cycle still synchronize at a period of 25.1h, as in (b). (d) As the strength of coupling of the circadian clock to the LD cycle increases, when parameter L varies as a square wave between 0 and v*_sPmax_* =1, the LD cycle can entrain both the circadian clock and the cell cycle to a period of 24h. (e) If the amplitude of the LD cycle is intermediate (L varies beween 0 and v*_sPmax_* =0.1), the intervals between peaks of the circadian clock and of the cell cycle vary between 24.6h and 25.2h. The curves are obtained for *V*_Cdk1_=12.59nM h^-1^, *v*_sw_=3.16μM h^-1^.

* * * * *

**11.** **Computer code for numerical simulations of the model for bidirectional coupling of cell cycle and circadian clock**

#circadian clock

Mp'=(v_sP*Bn^n/(K_AP^n+Bn^n)+v_in*KI_cdk1^ncdk/(KI_cdk1^ncdk+Mb^ncdk)-v_mP*Mp/(K_mP+Mp)-k_dmp*Mp)*delta

Mc'=(v_sC*Bn^n/(K_AC^n+Bn^n)+v_in*KI_cdk1^ncdk/(KI_cdk1^ncdk+Mb^ncdk)-v_mC*Mc/(K_mC+Mc)-k_dmc*Mc)*delta

Mbmal'=(v_sB*K_IB^m/(K_IB^m+Rn^m)+v_in*KI_cdk1^ncdk/(KI_cdk1^ncdk+Mb^ncdk)-v_mB*Mbmal/(K_mB+Mbmal)-k_dmb*Mbmal)*delta

Pc'=(k_sP*Mp-V_1P*Pc/(K_p+Pc)+V_2P*Pcp/(K_dp+Pcp)+k4_clock*PCc-k3_clock*Pc*Cc-k_dn*Pc)*delta

Cc'=(k_sC*Mc-V_1C*Cc/(K_p+Cc)+V_2C*Ccp/(K_dp+Ccp)+k4_clock*PCc-k3_clock*Pc*Cc-k_dnc*Cc)*delta

Pcp'=(V_1P*Pc/(K_p+Pc)-V_2P*Pcp/(K_dp+Pcp)-v_dPC*Pcp/(K_d+Pcp)-k_dn*Pcp)*delta

Ccp'=(V_1C*Cc/(K_p+Cc)-V_2C*Ccp/(K_dp+Ccp)-v_dCC*Ccp/(K_d+Ccp)-k_dn*Ccp)*delta

PCc'=(-V_1PC*PCc/(K_p+PCc)+V_2PC*PCcp/(K_dp+PCcp)-k4_clock*PCc+k3_clock*Pc*Cc+k2_clock*PCn-k1_clock*PCc-k_dn*PCc)*delta

PCn'=(-V_3PC*PCn/(K_p+PCn)+V_4PC*PCnp/(K_dp+PCnp)-k2_clock*PCn+k1_clock*PCc-k7*Bn*PCn+k8*In-k_dn*PCn)*delta

PCcp'=(V_1PC*PCc/(K_p+PCc)-V_2PC*PCcp/(K_dp+PCcp)-v_dPCC*PCcp/(K_d+PCcp)-k_dn*PCcp)*delta

PCnp'=(V_3PC*PCn/(K_p+PCn)-V_4PC*PCnp/(K_dp+PCnp)-v_dPCN*PCnp/(K_d+PCnp)-k_dn*PCnp)*delta

Bc'=(k_sB*Mbmal-(V_1B)*Bc/(K_p+Bc)+V_2B*Bcp/(K_dp+Bcp)-k5*Bc+k6*Bn-k_dn*Bc)*delta

Bcp'=((V_1B)*Bc/(K_p+Bc)-V_2B*Bcp/(K_dp+Bcp)-v_dBC*Bcp/(K_d+Bcp)-k_dn*Bcp)*delta

Bn'=(-V_3B*Bn/(K_p+Bn)+V_4B*Bnp/(K_dp+Bnp)+k5*Bc-k6*Bn-k7*Bn*PCn+k8*In-k_dn*Bn)*delta

Bnp'=(V_3B*Bn/(K_p+Bn)-V_4B*Bnp/(K_dp+Bnp)-v_dBN*Bnp/(K_d+Bnp)-k_dn*Bnp)*delta

In'=(-k8*In+k7*Bn*PCn-v_dIN*In/(K_d+In)-k_dn*In)*delta

Mr'=(v_sR*Bn^h/(K_AR^h+Bn^h)+v_in*KI_cdk1^ncdk/(KI_cdk1^ncdk+Mb^ncdk)-v_mR*Mr/(K_mR+Mr)-k_dmr*Mr)*delta

Rc'=(k_sR*Mr-k9*Rc+k10*Rn-(V_1R+V_cdk*Mb)*Rc/(K_p+Rc)+V_2R*Rcp/(K_dp+Rcp)-k_dn*Rc)*delta

Rcp'=((V_1R+V_cdk*Mb)*Rc/(K_p+Rc)-V_2R*Rcp/(K_dp+Rcp)-v_dRC*Rcp/(K_d+Rcp)-k_dn*Rcp)*delta

Rn'=(k9*Rc-k10*Rn-(V_3R+V_cdk*Mb)*Rn/(K_p+Rn)+V_4R*Rnp/(K_dp+Rnp)-k_dn*Rn)*delta

Rnp'=((V_3R+V_cdk*Mb)*Rn/(K_p+Rn)-V_4R*Rnp/(K_dp+Rnp)-v_dRN*Rnp/(K_d+Rnp)-k_dn*Rnp)*delta

#cell cycle

# Mitotic stimulation by growth factor, GF

AP1'=(v_sap1*(GF/(K_agf+GF))-k_dap1*AP1)*eps

# Antagonistic regulation exerted by pRB and E2F

pRB'=(v_sprb-k_pc1*pRB*E2F+k_pc2*pRBc1-V1*(pRB/(K1+pRB))*(Md+Mdp27)+V2*(pRBp/(K2+pRBp))-k_dprb*pRB)*eps

pRBc1'=(k_pc1*pRB*E2F-k_pc2*pRBc1)*eps

pRBp'=(V1*(pRB/(K1+pRB))*(Md+Mdp27)-V2*(pRBp/(K2+pRBp))-V3*(pRBp/(K3+pRBp))*Me+V4*(pRBpp/(K4+pRBpp))-k_pc3*pRBp*E2F+k_pc4*pRBc2-k_dprbp*pRBp)*eps

pRBc2'=(k_pc3*pRBp*E2F-k_pc4*pRBc2)*eps

pRBpp'=(V3*(pRBp/(K3+pRBp))*Me-V4*(pRBpp/(K4+pRBpp))-k_dprbpp*pRBpp)*eps

E2F'=(v_se2f-k_pc1*pRB*E2F+k_pc2*pRBc1-k_pc3*pRBp*E2F+k_pc4*pRBc2-V_1e2f*Ma*(E2F/(K_1e2f+E2F))+V_2e2f*(E2Fp/(K_2e2f+E2Fp))-k_de2f*E2F)*eps

E2Fp'=(V_1e2f*Ma*(E2F/(K_1e2f+E2F))-V_2e2f*(E2Fp/(K_2e2f+E2Fp))-k_de2fp*E2Fp)*eps

# Module Cyclin D/Cdk4-6 : G1 phase

Cd'=(k_cd1*AP1+k_cd2*E2F*(K_i7/(K_i7+pRB))*(K_i8/(K_i8+pRBp))-k_com1*Cd*(Cdk4_tot-(Mdi+Md+Mdp27))+k_decom1*Mdi-V_dd*(Cd/(K_dd+Cd))-k_ddd*Cd)*eps

Mdi'=(k_com1*Cd*(Cdk4_tot-(Mdi+Md+Mdp27))-k_decom1*Mdi+V_m2d*(Md/(K_2d+Md))-V_m1d*(Mdi/(K_1d+Mdi)))*eps

Md'=(V_m1d*(Mdi/(K_1d+Mdi))-V_m2d*(Md/(K_2d+Md))-k_c1*Md*p27+k_c2*Mdp27)*eps

Mdp27'=(k_c1*Md*p27-k_c2*Mdp27)*eps

#Module Cyclin E/Cdk2: G1 phase and transition G1/S

Mce'=v_sce*K_ice^nce/(K_ice^nce+Bn^nce)-V_dmce*Mce/(K_dmce+Mce)

Ce'=(k_ce*E2F*(K_i9/(K_i9+pRB))*(K_i10/(K_i10+pRBp))+k_ce2*Mce-k_com2*Ce*(Cdk2_tot-(Mei+Me+Mep27+Mai+Ma+Map27))+k_decom2*Mei-V_de*(Skp2/(K_dceskp2+Skp2))*(Ce/(K_de+Ce))-k_dde*Ce)*eps

Mei'=(k_com2*Ce*(Cdk2_tot-(Mei+Me+Mep27+Mai+Ma+Map27))-k_decom2*Mei+V_m2e*(Wee

1+i_b1)*(Me/(K_2e+Me))-V_m1e*Pe*(Mei/(K_1e+Mei)))*eps

Me'=(V_m1e*Pe*(Mei/(K_1e+Mei))-V_m2e*(Wee1+i_b1)*(Me/(K_2e+Me))-k_c3*Me*p27+k_c4*Mep27)*eps

Skp2'=(v_sskp2-V_dskp2*(Skp2/(K_dskp2+Skp2))*(Cdh1a/(K_cdh1+Cdh1a))-k_ddskp2*Skp2)*eps

Mep27'=(k_c3*Me*p27-k_c4*Mep27)*eps

Pei'=(v_spei+V_6e*(x_e1+x_e2*Chk1)*(Pe/(K_6e+Pe))-V_m5e*(Me+a_e)*(Pei/(K_5e+Pei))-k_dpei*Pei)*eps

Pe'=(V_m5e*(Me+a_e)*(Pei/(K_5e+Pei))-V_6e*(x_e1+x_e2*Chk1)*(Pe/(K_6e+Pe))-k_dpe*Pe)*eps

# Module Cyclin A/Cdk2 : S phase and transition S/G2

Ca'=(k_ca*E2F*(K_i11/(K_i11+pRB))*(K_i12/(K_i12+pRBp))-k_com3*Ca*(Cdk2_tot-(Mei+Me+Mep27+Mai+Ma+Map27))+k_decom3*Mai-V_da*(Ca/(K_da+Ca))*(Cdc20a/(K_acdc20+Cdc20a))-k_dda*Ca)*eps

Mai'=(k_com3*Ca*(Cdk2_tot-(Mei+Me+Mep27+Mai+Ma+Map27))-k_decom3*Mai+V_m2a*(Wee1+i_b2)*(Ma/(K_2a+Ma))-V_m1a*Pa*(Mai/(K_1a+Mai)))*eps

Ma'=(V_m1a*Pa*(Mai/(K_1a+Mai))-V_m2a*(Wee1+i_b2)*(Ma/(K_2a+Ma))-k_c5*Ma*p27+k_c6*Map27)*eps

Map27'=(k_c5*Ma*p27-k_c6*Map27)*eps

p27'=(v_s1p27+v_s2p27*E2F*(K_i13/(K_i13+pRB))*(K_i14/(K_i14+pRBp))-k_c1*Md*p27+k_c2*Mdp27-k_c3*Me*p27+k_c4*Mep27-k_c5*Ma*p27+k_c6*Map27-k_c7*Mb*p27+k_c8*Mbp27-V_1p27*Me*(p27/(K_1p27+p27))+V_2p27*(p27p/(K_2p27+p27p))-k_ddp27*p27)*eps

p27p'=(V_1p27*Me*(p27/(K_1p27+p27))-V_2p27*(p27p/(K_2p27+p27p))-V_dp27p*(Skp2/(K_dp27skp2+Skp2))*(p27p/(K_dp27p+p27p))-k_ddp27p*p27p)*eps

Cdh1i'=(V_2cdh1*(Cdh1a/(K_2cdh1+Cdh1a))*(Ma+Mb)-V_1cdh1*(Cdh1i/(K_1cdh1+Cdh1i))-k_dcdh1i*Cdh1i)*eps

Cdh1a'=(v_scdh1a+V_1cdh1*(Cdh1i/(K_1cdh1+Cdh1i))-V_2cdh1*(Cdh1a/(K_2cdh1+Cdh1a))*(Ma+Mb)-k_dcdh1a*Cdh1a)*eps

Pai'=(v_spai+V_6a*(x_a1+x_a2*Chk1)*(Pa/(K_6a+Pa))-V_m5a*(Ma+a_a)*(Pai/(K_5a+Pai))-k_dpai*Pai)*eps

Pa'=(V_m5a*(Ma+a_a)*(Pai/(K_5a+Pai))-V_6a*(x_a1+x_a2*Chk1)*(Pa/(K_6a+Pa))-k_dpa*Pa)*eps

# Module Cyclin B/Cdk1 : G2 phase and transition G2/M

Cb'=(v_cb-k_com4*Cb*(Cdk1_tot-(Mbi+Mb+Mbp27))+k_decom4*Mbi-V_db*(Cb/(K_db+Cb))*((Cdc20a/(K_dbcdc20+Cdc20a))+(Cdh1a/(K_dbcdh1+Cdh1a)))-k_ddb*Cb)*eps

Mbi'=(k_com4*Cb*(Cdk1_tot-(Mbi+Mb+Mbp27))-k_decom4*Mbi+V_m2b*(Wee1+i_b3)*(Mb/(K_2b+Mb))-V_m1b*Pb*(Mbi/(K_1b+Mbi)))*eps

Mb'=(V_m1b*Pb*(Mbi/(K_1b+Mbi))-V_m2b*(Wee1+i_b3)*(Mb/(K_2b+Mb))-k_c7*Mb*p27+k_c8*Mbp27)*eps

Mbp27'=(k_c7*Mb*p27-k_c8*Mbp27)*eps

Cdc20i'=(v_scdc20i-V_m3b*Mb*(Cdc20i/(K_3b+Cdc20i))+V_m4b*(Cdc20a/(K_4b+Cdc20a))-k_dc

dc20i*Cdc20i)*eps

Cdc20a'=(V_m3b*Mb*(Cdc20i/(K_3b+Cdc20i))-V_m4b*(Cdc20a/(K_4b+Cdc20a))-k_dcdc20a*Cdc20a)*eps

Pbi'=(v_spbi+V_6b*(x_b1+x_b2*Chk1)*(Pb/(K_6b+Pb))-V_m5b*(Mb+a_b)*(Pbi/(K_5b+Pbi))-k_dpbi*Pbi)*eps

Pb'=(V_m5b*(Mb+a_b)*(Pbi/(K_5b+Pbi))-V_6b*(x_b1+x_b2*Chk1)*(Pb/(K_6b+Pb))-k_dpb*Pb)*eps

#coupling via wee1

Mw'=v_swee1+v_sw*Bn^nmw/(K_aw^nmw+Bn^nmw)-V_dmw*Mw/(K_dmw+Mw)

Wee1'=(k_sw*Mw-V_m7b*(Mb+i_b)*(Wee1/(K_7b+Wee1))+V_m8b*(Wee1p/(K_8b+Wee1p))-k_dwee1*Wee1)*eps

Wee1p'=(V_m7b*(Mb+i_b)*(Wee1/(K_7b+Wee1))-V_m8b*(Wee1p/(K_8b+Wee1p))-k_dwee1p*Wee1p)*eps

#parameters

par V_cdk=3.1623,KI_cdk1=0.5,ncdk=2

par v_sw=0.1

par k1_clock=0.8,k2_clock=0.4,k3_clock=0.8,k4_clock=0.4,k5=0.8,k6=0.4,k7=1,k8=0.2,k9=0.63,k10=0.4,\

K_AP=0.6,K_AC=0.6,K_AR=0.6,K_IB=1,\

k_dmb=0.02,k_dmc=0.02,k_dmp=0.02,k_dmr=0.02,k_dn=0.02,k_dnc=0.02,\

K_d=0.3,K_dp=0.1,K_p=1.006,K_mB=0.4,K_mC=0.4,K_mP=0.3,K_mR=0.4,k_sB=0.32,\

k_sC=3.2,k_sP=1.2,k_sR=1.7,m=2,h=2,n=2,\

V_1B=1.4,V_1C=1.2,V_1P=9.6,V_1PC=2.4,V_2B=0.2,V_2C=0.2,V_2P=0.6,V_2PC=0.2,\

V_3B=1.4,V_3PC=2.4,V_4B=0.4,V_4PC=0.2,V_phos=0.4,v_dBC=3,v_dBN=3,v_dCC=1.4,\

v_dIN=1.6,v_dPC=3.4,v_dPCC=1.4,v_dPCN=1.4,v_dRC=4.4,v_dRN=0.8,v_mB=1.3,\

v_mC=2.0,v_mP=2.2,v_mR=1.6,v_sB=1.8,\

v_sC=2.2,v_sP=2.4,v_sR=1.6,\

V_1R=4,V_2R=8,V_3R=8,V_4R=4,v_in=0.7

par delta=1

par Chk1=0

par GF=1,K_agf=0.1,k_dap1=0.15,eps=21.58,v_sap1=1

par k_de2f=0.002,k_de2fp=1.1,k_dprb=0.01,k_dprbp=0.06,k_dprbpp=0.04

par k_pc1=0.05,k_pc2=0.5,k_pc3=0.025,k_pc4=0.5,K1=0.1,K2=0.1,K3=0.1

par K4=0.1,V1=2.2,V2=2,V3=1,V4=2,K_1e2f=5,K_2e2f=5,V_1e2f=4

par V_2e2f=0.75,v_se2f=0.15,v_sprb=0.8

par Cdk4_tot=1.5,K_i7=0.1,K_i8=2,k_cd1=0.4,k_cd2=0.005,k_decom1=0.1

par k_com1=0.175,k_c1=0.15,k_c2=0.05,k_ddd=0.005,K_dd=0.1,K_1d=0.1,K_2d=0.1

par V_dd=5,V_m1d=1,V_m2d=0.2

par a_e=0.25,Cdk2_tot=2,i_b1=0.5,K_i9=0.1,K_i10=2,k_ce=0.29,k_c3=0.2

par k_c4=0.1,k_decom2=0.1,k_com2=0.2,k_dde=0.005,k_ddskp2=0.005,k_dpe=0.075

par k_dpei=0.15,K_de=0.1,K_dceskp2=2,K_dskp2=0.5,K_cdh1=0.4,K_1e=0.1

par K_2e=0.1,K_5e=0.1,K_6e=0.1,V_de=3,V_dskp2=1.1,V_m1e=2,V_m2e=1.4,V_m5e=5

par V_6e=0.8,v_spei=0.13,v_sskp2=0.15,x_e1=1,x_e2=1

par a_a=0.2,i_b2=0.5,K_i11=0.1,K_i12=2,K_i13=0.1,K_i14=2,k_ca=0.0375

par k_decom3=0.1,k_com3=0.2,k_c5=0.15,k_c6=0.125,k_dda=0.005,k_ddp27=0.06

par k_ddp27p=0.01,k_dcdh1a=0.1,k_dcdh1i=0.2,k_dpa=0.075,k_dpai=0.15,K_da=1.1

par K_dp27p=0.1,K_dp27skp2=0.1,K_acdc20=2,K_1a=0.1,K_2a=0.1,K_1cdh1=0.01

par K_2cdh1=0.01,K_5a=0.1,K_6a=0.1,K_1p27=0.5,K_2p27=0.5,V_dp27p=5,V_da=2.5

par V_m1a=2,V_m2a=1.85,V_m5a=4,V_6a=1,v_scdh1a=0.11,v_spai=0.105

par v_s1p27=0.8,v_s2p27=0.1,V_1cdh1=1.25,V_2cdh1=8,V_1p27=100,V_2p27=0.1

par x_a1=1,x_a2=1

par a_b=0.11,Cdk1_tot=0.5,i_b=0.75,i_b3=0.5,k_c7=0.12,k_c8=0.2

par k_decom4=0.1,k_com4=0.25,k_dcdc20a=0.05,k_dcdc20i=0.14,k_ddb=0.005

par k_dpb=0.1,k_dpbi=0.2,k_dwee1=0.1,k_dwee1p=0.2,K_db=0.005,K_dbcdc20=0.2,K_dbcdh1=0.1

par k_sw=5,K_1b=0.1,K_2b=0.1,K_3b=0.1,K_4b=0.1,K_5b=0.1,K_6b=0.1,K_7b=0.1

par K_8b=0.1,v_cb=0.055,V_db=0.06,V_m1b=3.9,V_m2b=2.1,v_scdc20i=0.1,V_m3b=8,V_m4b=0.7

par V_m5b=5,V_6b=1,V_m7b=1.2,V_m8b=1,v_spbi=0.12,x_b1=1,x_b2=1

par v_swee1=0.0117,nmw=4,K_aw=2,V_dmw=0.5,K_dmw=0.5

par v_sce=0.005,K_ice=1,V_dmce=0.5,K_dmce=0.5,nce=4,k_ce2=5

# initial conditions

init Mp=0.1,Mc=0.1,Mbmal=0.1,Pc=0.1,Cc=0.1,\

Pcp=0.1,Ccp=0.1,PCc=0.1,PCn=0.1,PCcp=0.1

init PCnp=0.1,Bc=0.1,Bcp=0.1,Bn=0.1,Bnp=0.1,\

In=0.1,Mr=0.1,Rc=0.1,Rcp=0.1,Rn=0.1,Rnp=0.1

init AP1=0.01,pRB=1,pRBc1=0.25,pRBp=0.1,pRBc2=0.01,pRBpp=0.01,E2F=0.1,E2Fp=0.05

init Cd=0.01,Mdi=0.01,Md=0.01,Mdp27=0.01

init Mce=0.1,Ce=0.01,Mei=0.01,Me=0.01,Skp2=0.01,Mep27=0.01,Pei=0.01,Pe=0.01

init Ca=0.01,Mai=0.01,Ma=0.01,Map27=0.01,p27=0.25,p27p=0.01,Cdh1i=0.01,Cdh1a=0.01,Pai=0.01,Pa=0.01

init Cb=0.01,Mbi=0.01,Mb=0.01,Mbp27=0.01,Cdc20i=0.01,Cdc20a=0.01,Pbi=0.01,Pb=0.01,Mw=0,Wee1=0.1

init Wee1p=0.01

@ bounds=1000000,total=1000,Method=Qualst.RK4,Dt=0.01

@ runnow=1

Done

*** * * * ***

The following lines of code must be used when simulating in Fig. 11 the coupling of the circadian clock to the cell cycle through putative phosphorylation of BMAL1 by CDK1:

Bc'=(k_sB*Mbmal-(V_1B+V_cdk*Mb)*Bc/(K_p+Bc)+V_2B*Bcp/(K_dp+Bcp)-k5*Bc+k6*Bn-k_dn*Bc)*delta

Bcp'=((V_1B+V_cdk*Mb)*Bc/(K_p+Bc)-V_2B*Bcp/(K_dp+Bcp)-v_dBC*Bcp/(K_d+Bcp)-k_dn*Bcp)*delta

Bn'=(-V_3B*Bn/(K_p+Bn)+V_4B*Bnp/(K_dp+Bnp)+k5*Bc-k6*Bn-k7*Bn*PCn+k8*In-k_dn*Bn)*delta

Bnp'=(V_3B*Bn/(K_p+Bn)-V_4B*Bnp/(K_dp+Bnp)-v_dBN*Bnp/(K_d+Bnp)-k_dn*Bnp)*delta

In'=(-k8*In+k7*Bn*PCn-v_dIN*In/(K_d+In)-k_dn*In)*delta

Mr'=(v_sR*Bn^h/(K_AR^h+Bn^h)-v_mR*Mr/(K_mR+Mr)-k_dmr*Mr)*delta

Rc'=(k_sR*Mr-k9*Rc+k10*Rn-v_dRC*Rc/(K_d+Rc)-k_dn*Rc)*delta

Rn'=(k9*Rc-k10*Rn-v_dRN*Rn/(K_d+Rn)-k_dn*Rn)*delta

The following lines of code must be deleted when simulating in Fig. 11 the coupling of the circadian clock to the cell cycle through putative phosphorylation of BMAL1 by CDK1:

Bc'=(k_sB*Mbmal-(V_1B)*Bc/(K_p+Bc)+V_2B*Bcp/(K_dp+Bcp)-k5*Bc+k6*Bn-k_dn*Bc)*delta

Bcp'=((V_1B)*Bc/(K_p+Bc)-V_2B*Bcp/(K_dp+Bcp)-v_dBC*Bcp/(K_d+Bcp)-k_dn*Bcp)*delta

Bn'=(-V_3B*Bn/(K_p+Bn)+V_4B*Bnp/(K_dp+Bnp)+k5*Bc-k6*Bn-k7*Bn*PCn+k8*In-k_dn*Bn)*delta

Bnp'=(V_3B*Bn/(K_p+Bn)-V_4B*Bnp/(K_dp+Bnp)-v_dBN*Bnp/(K_d+Bnp)-k_dn*Bnp)*delta

In'=(-k8*In+k7*Bn*PCn-v_dIN*In/(K_d+In)-k_dn*In)*delta

Mr'=(v_sR*Bn^h/(K_AR^h+Bn^h)+v_in*KI_cdk1^ncdk/(KI_cdk1^ncdk+Mb^ncdk)-v_mR*Mr/(K_mR+Mr)-k_dmr*Mr)*delta

Rc'=(k_sR*Mr-k9*Rc+k10*Rn-(V_1R+V_cdk*Mb)*Rc/(K_p+Rc)+V_2R*Rcp/(K_dp+Rcp)-k_dn*Rc)*delta

Rcp'=((V_1R+V_cdk*Mb)*Rc/(K_p+Rc)-V_2R*Rcp/(K_dp+Rcp)-v_dRC*Rcp/(K_d+Rcp)-k_dn*Rcp)*delta

Rn'=(k9*Rc-k10*Rn-(V_3R+V_cdk*Mb)*Rn/(K_p+Rn)+V_4R*Rnp/(K_dp+Rnp)-k_dn*Rn)*delta

Rnp'=((V_3R+V_cdk*Mb)*Rn/(K_p+Rn)-V_4R*Rnp/(K_dp+Rnp)-v_dRN*Rnp/(K_d+Rnp)-k_dn*Rnp)*delta

* * * * *

**12. References cited in Supporting Informations (SI Refs)**

1. Leloup JC, Goldbeter A. 2003 Toward a detailed computational model for the mammalian circadian clock. *Proc. Natl. Acad. Sci. USA* **100**, 7051-7056.

2. Gérard C, Goldbeter A. 2009 Temporal self-organization of the cyclin/Cdk network driving the mammalian cell cycle. *Proc. Natl. Acad. Sci. USA* **106**, 21643-21648.

3. Gérard C, Goldbeter A. 2012 Entrainment of the mammalian cell cycle by the circadian clock: Modeling two coupled cellular rhythms. *PLoS Comput. Biol.* **8**, e1002516.

4. Morgan DO. 2006 *The cell cycle: Principles of control.* Oxford, UK: Oxford Univ. Press.

5. Matsuo T, Yamaguchi S, Mitsui S, Emi A, Shimoda F, Okamura H. 2003 Control mechanism of the circadian clock for timing of cell division in vivo. *Science* **302**, 255-259.

6. Zhao X, Hirota T, Han X, Cho H, Chong LW, Lamia K, et al. 2016 Circadian amplitude regulation via FBXW7-targeted REV-ERBα degradation. *Cell* **165**, 1644–1657.

7. Pérez-Roger I, Solomon DLC, Sewing A, Land H. 1997 Myc activation of cyclin E/Cdk2 kinase involves induction of cyclin E gene transcription and inhibition of p27^Kip1^ binding to newly formed complexes. *Oncogene* **14**, 2373-2381.

8. Fu L, Pelicano H, Liu J, Huang P, Chi Lee C. 2002 The circadian gene *Period2* plays an important role in tumor suppression and DNA damage response in vivo. *Cell* **111**, 41-50.

9. Bieler J, Cannavo R, Gustafson K, Gobet, C, Gatfield D, Naef F. 2014 Robust synchronization of coupled circadian and cell cycle oscillators in single mammalian cells. *Mol. Syst Biol.* **10,** 739. (doi: 10.15252/msb.20145218.)

10. Gottesfeld JM, Forbes DJ. 1997 Mitotic repression of the transcriptional machinery. *Trends Biochem. Sci.* **22**, 197-202.

11. Goldbeter A, Koshland DE Jr. 1981 An amplified sensitivity arising from covalent modification in biological systems. *Proc. Natl. Acad. Sci. USA* **78**, 6840-6844.

12. Zylka MJ, Shearman LP, Weaver DR, Reppert SM. 1998 Three *period* homologs in mammals: differential light responses in the suprachiasmatic circadian clock and oscillating transcripts outside of brain. *Neuron* **20**, 1103–1110.

13. Kwak Y, Jeong J, Lee S, Park YU, Lee SA, Han DH, Kim JH, Ohshima T, Mikoshiba K, Suh YH, Cho S, Park SK. 2013 Cyclin-dependent kinase 5 (Cdk5) regulates the function of CLOCK protein by direct phosphorylation. *J. Biol. Chem.* **288**, 36878-36889.

14. Ermentrout B. 2007 XPPAUT. *Scholarpedia* **2**(1), 1399.

* * * * *
